# Supplementary material for: Functional tests of the competitive exclusion hypothesis for multituberculate extinction
Source: R Soc Open Sci. 2019 Mar 27;6(3):181536. doi: 10.1098/rsos.181536 (PMC6458384; doi:10.1098/rsos.181536)
Supplement: Supplementary methods and results [file rsos181536supp1.pdf]

Supplemental material for:

Functional tests of the competitive exclusion hypothesis for multituberculate mammal extinction

Neil F. Adams, Emily J. Rayfield, Philip G. Cox, Samuel N. Cobb, Ian J. Corfe

Contents

1. Supplementary material and methods **2**
(a) Calculating masticatory muscle forces 2
(b) Finite element analysis 7
(i) FE meshes and material properties 7
(ii) Model constraints 9
(iii) Input muscle forces and scaling of DigiMorph specimens 10
(c) Calculating bite force quotient (BFQ) 11
(d) Biomechanical analysis in MomentMacro 11
(e) Shapiro-Wilk tests of normality for Z_x, Z_y and J data 20
2. Supplementary results **22**
(a) Stress distribution supplementary figures 22
(b) Cox *et al*. (2012) cranial stress distribution figures 31
(c) Average stress data 32
(d) Mechanical efficiency data 34
(e) Z_x, Z_y, J supplementary data 35
References **36**

1. Supplementary material and methods

(a) Calculating masticatory muscle forces

Muscle forces have been previously calculated for the squirrel, guinea pig and rat [1] following the 'dry skull' method of Thomason [2], which uses muscle cross-sectional area to determine force. To calculate the muscle force for the mouse (Table S1) and *Kryptobaatar* (Table S2), volumes of each muscle were measured using the Label Analysis module in Avizo. Approximations of muscle fibre lengths were made using the Avizo measurement tool. Ten measurements were made for each muscle between the origin and attachment sites and were then averaged to provide a mean fibre length. This assumes that muscle fibre length is equal to the length of the entire muscle, which may not be the case in reality, but this approach follows that used for extant rodents [1], which showed that similar digital estimates were within 1.5 mm of *ex vivo* measurements. Physiological cross-sectional areas (PCSAs) were calculated by dividing the muscle volume by the mean fibre length, and muscle forces were calculated by multiplying PCSAs by an isometric stress value of 0.3 N/mm^2 [1, 2].

The 3D volumes of masticatory muscles of extant rodents are known and can be precisely measured. However, reconstructing 3D muscles for fossil taxa lacking soft tissues, such as *Kryptobaatar*, is problematic. While identifying muscle origin and attachment sites was relatively straightforward due to the excellent preservation of the *Kryptobaatar* skull, the final 'fleshing-out' stage required more inference. For some muscles, such as the intermediate zygomaticomandibularis and deep masseter, the final 3D shape was tightly constrained by available space between bones and surrounding muscles. Other muscles were expanded to fill available spaces, such as the filling of the temporal fenestra by the temporalis muscles. The

most subjectivity was introduced by determining the thickness of outwardly unconstrained muscles, such as the superficial masseters and anterior zygomaticomandibularis. To minimize uninformed inference, the minimum distance required to connect origin and insertion sites was reconstructed. This could lead to underestimations of muscle volume and forces if muscles thickened significantly between origin and attachment sites. However, this cannot be assessed from osteological correlates and would require high levels of speculation.

Table S1. Volumes (from [3]), physiological cross-sectional areas (PCSAs) and forces of masticatory muscles for *Mus musculus*.

**Muscle**	**Volume (mm^3)**	**% of total muscle volume**	**Mean fibre length (mm)**	**PCSA (mm^2)**	**Muscle force (N)**	**% of total muscle force**
Deep masseter	101.89	34.01	5.16	19.73	5.92	28.26
Superficial masseter	52.83	17.63	6.07	8.70	2.61	12.46
Temporalis	68.63	22.91	4.88	14.05	4.22	20.13
External pterygoid	14.49	4.84	1.66	8.72	2.61	12.49
Internal pterygoid	35.38	11.81	3.43	10.33	3.10	14.80
Infraorbital zygomatico-mandibularis	7.17	2.39	4.43	1.62	0.49	2.32
Anterior zygomatico-mandibularis	15.71	5.24	3.25	4.84	1.45	6.93
Posterior zygomatico-mandibularis	3.52	1.17	1.93	1.82	0.55	2.61
**Total**	**299.62**	**100.00**			**20.94**	**100.00**

Table S2. Volumes, physiological cross-sectional areas (PCSAs) and forces of masticatory muscles for *Kryptobaatar dashzevegi*.

**Muscle**	**Volume (mm^3)**	**% of total muscle volume**	**Mean fibre length (mm)**	**PCSA (mm^2)**	**Muscle force (N)**	**% of total muscle force**
Deep masseter	45.87	16.13	6.05	7.58	2.27	17.43
Anterior superficial masseter	24.61	8.65	7.72	3.19	0.96	7.32
Posterior superficial masseter	8.67	3.05	6.27	1.38	0.41	3.18
Medial temporalis	49.60	17.44	9.70	5.11	1.53	11.75
Lateral temporalis	71.29	25.07	6.72	10.61	3.18	24.38
External pterygoid	10.98	3.86	3.65	3.01	0.90	6.92
Internal pterygoid	25.97	9.13	4.35	5.97	1.79	13.71
Anterior zygomatico-mandibularis	30.01	10.55	8.46	3.55	1.06	8.15
Intermediate zygomatico-mandibularis	15.87	5.58	5.85	2.71	0.81	6.23
Posterior zygomatico-mandibularis	1.50	0.53	3.71	0.40	0.12	0.93
**Total**	**284.37**	**100.00**			**13.06**	**100.00**

Figure S1. Three-dimensional masticatory muscle reconstructions for *Kryptobaatar dashzevegi* (based on muscle reconstructions by [4]). The anterior and posterior superficial masseter (a), deep masseter (b), anterior, intermediate and posterior zygomaticomandibularis (c), and anterior and posterior temporalis (d) are shown in left lateral view. The internal and external pterygoids (e) are shown in dorsally oblique, right lateral view.

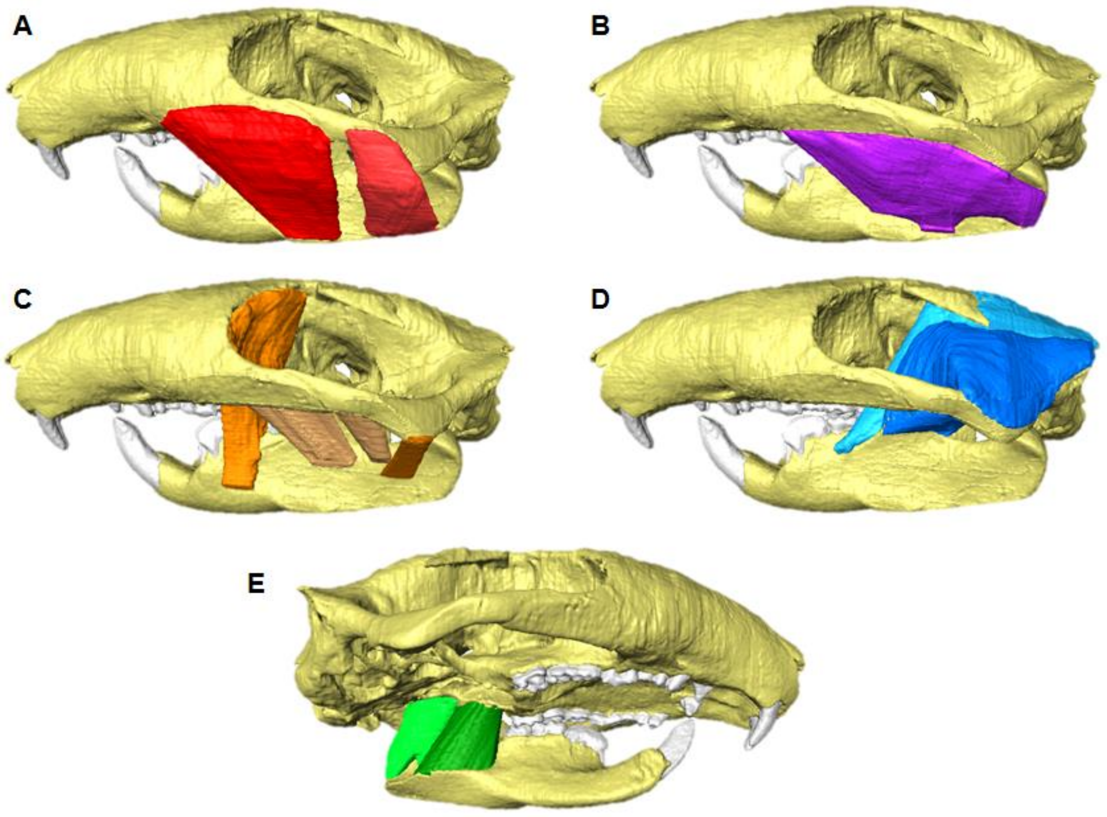

Figure S2. Muscle attachment sites on the hemimandibles of *(a-c) Rattus*, *(d-f) Sciurus*, and *(g-i) Cavia*. *(a), (d)* and *(g)* in lateral view, *(b), (e) and (h)* in medial view, and *(c), (f)* and *(i)* in occlusal/dorsal view. Attachment sites based on muscle models from [1, 5].

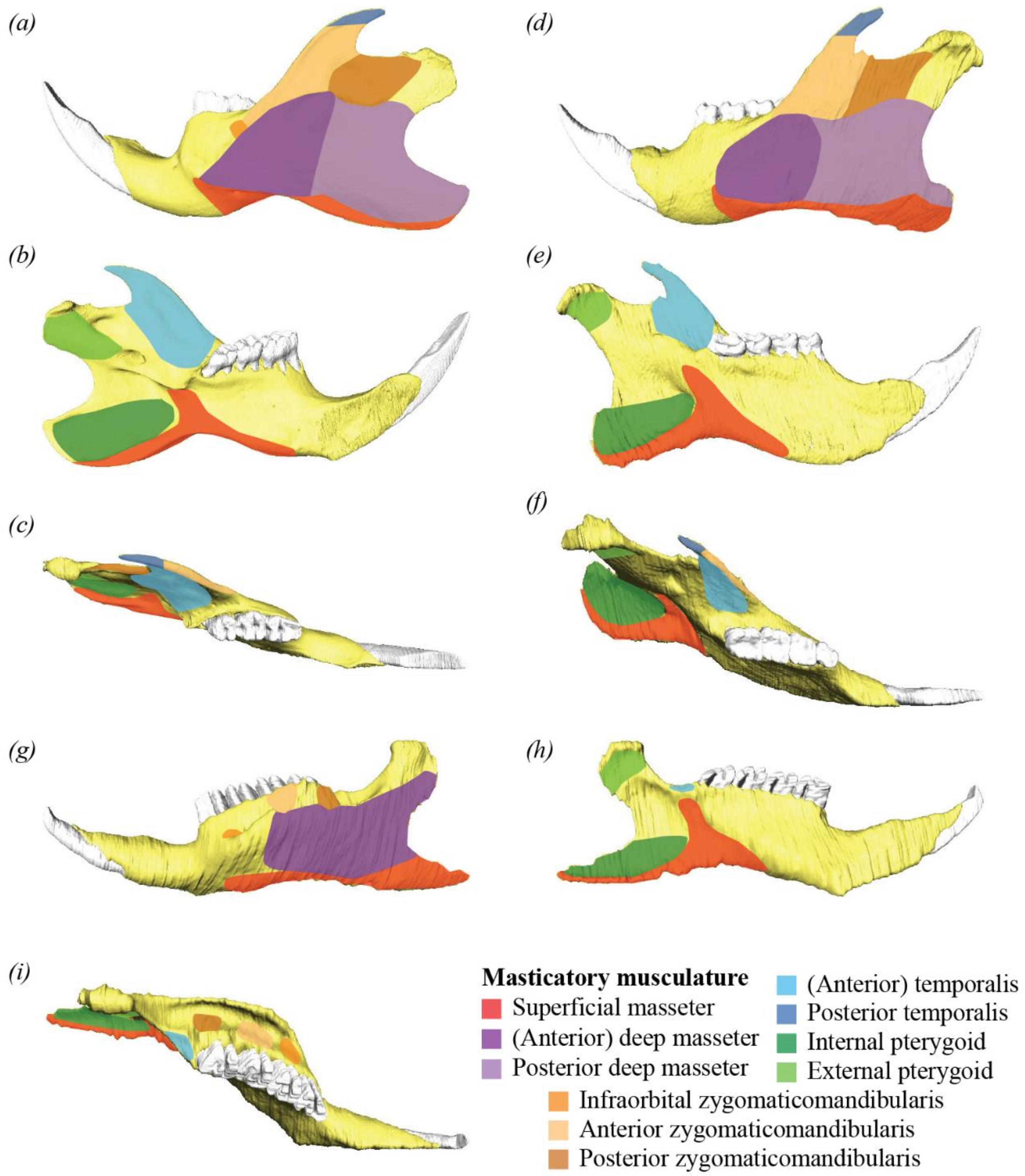

(b) Finite element analysis

(i) FE meshes and material properties

The digital models of the hemimandibles and crania were converted into 3D meshes using HyperMesh 14.0 (Altair Engineering Inc., Troy, MI, USA). The hemimandible meshes were composed of between 115,000 and 142,000 tetrahedral linear elements. The cranial meshes for *Kryptobaatar* and the mouse were made up of 670,000 and 1,210,000 elements, respectively. All meshes had an average element size of 0.25 mm. This element size was chosen to replicate that used by Cox *et al*. [1], who used this value because it was well under 0.92 mm; the value at which FEA results were found to converge for a pig skull [6]. Further details of each mesh are provided below in Tables S3-S4.

Table S3. Details of finite element meshes made for the rodent and *Kryptobaatar* hemimandibles, and calculation of muscle to bone volume ratios.

Specimen	Elements	Nodes	Hemimandible length (mm)	Hemimandible width (mm)	HyperMesh volume (mm^3)	Total muscle volume (mm^3)	Muscle: bone ratio
DigiMorph mouse	119659	25800	14.9	5.7	34.99	299.62	8.563
DigiMorph rat	125789	26224	33.1	10.0	342.52	2451.76	7.158
Guinea pig	115487	23905	43.8	21.0	1309.49	3674.39	2.806
Squirrel	119132	25599	39.7	12.0	752.48	3405.71	4.526
Kryptobaatar	142262	29599	18.3	7.8	72.22	284.37	3.938

Table S4. Details of finite element meshes made for the mouse and *Kryptobaatar* crania, and calculation of muscle to bone volume ratios.

Specimen	Elements	Nodes	Skull length (mm)	Skull width (mm)	HyperMesh volume (mm^3)	Total muscle volume (mm^3)	Muscle: bone ratio
DigiMorph mouse	1214131	253112	24.9	13.0	199.40	599.24	3.005
Kryptobaatar	672954	159827	27.3	20.0	474.99	568.74	1.197

Each component of the mesh (bone, premolars-molars, and incisors) was modelled as linearly elastic, and isotropic, homogeneous material properties were applied to the components based on measured values from rodents [1]: a Young's modulus of 18 GPa for bone, 30 GPa for premolars-molars, 70 GPa for incisors, and a Poisson's ratio of 0.3 for all materials.

The effects of varying these material properties were explored in sensitivity analyses, to account for the fact that material properties cannot be validated for lithified fossils. Six analyses were undertaken for each bite point in the *Kryptobaatar* models (Tables S5-S7). In three tests (1, 3 and 5) the Poisson's ratio was kept constant at 0.3 and the Young's modulus was varied using low, intermediate and high estimates. In tests 2, 4 and 6 the Poisson's ratio was raised to 0.33. The values chosen were based on similar sensitivity analyses of rodent skulls by Cox *et al*. [7]. As shown in Tables S6-S7, differences in average stress values were minimal despite changes in material properties.

Table S5. Material properties used in the six sensitivity analyses of the *Kryptobaatar* hemimandible and cranium finite element models.

**Test number**	**Young's Modulus (GPa)**			**Poisson's ratio**
	**Bone**	**Premolars-Molars**	**Incisors**	
1	10	20	15	0.3
2	10	20	15	0.33
3	20	30	50	0.3
4	20	30	50	0.33
5	30	40	80	0.3
6	30	40	80	0.33

Table S6. Median von Mises stress values (in MPa) from the sensitivity tests of the *Kryptobaatar* hemimandible, showing maximum and minimum values and the difference between them.

	**Test 1**	**Test 2**	**Test 3**	**Test 4**	**Test 5**	**Test 6**	**Max**	**Min**	**Diff.**
**i1**	3.020	3.012	3.020	3.010	3.008	2.999	3.020	2.999	0.021
**p4**	1.316	1.313	1.370	1.367	1.376	1.373	1.376	1.313	0.063
**m1**	1.194	1.189	1.234	1.229	1.239	1.234	1.239	1.189	0.050
**m2**	1.498	1.487	1.624	1.612	1.657	1.646	1.657	1.487	0.170

Table S7. Median von Mises stress values (in MPa) from the sensitivity tests of the *Kryptobaatar* cranium, showing maximum and minimum values and the difference between them.

	**Test 1**	**Test 2**	**Test 3**	**Test 4**	**Test 5**	**Test 6**	**Max**	**Min**	**Diff.**
**I2**	1.123	1.121	1.123	1.120	1.123	1.120	1.123	1.120	0.003
**I3**	1.051	1.049	1.047	1.045	1.045	1.043	1.051	1.043	0.008
**P1**	0.889	0.887	0.887	0.884	0.886	0.883	0.889	0.883	0.006
**P2**	0.860	0.858	0.858	0.855	0.857	0.854	0.860	0.854	0.006
**P3**	0.800	0.798	0.798	0.795	0.797	0.794	0.800	0.794	0.006
**P4**	0.716	0.713	0.715	0.712	0.714	0.711	0.716	0.711	0.005
**M1**	0.632	0.629	0.629	0.627	0.628	0.625	0.632	0.625	0.007

(ii) Model constraints

The mouse and *Kryptobaatar* cranial models were constrained in a comparable way to the cranial models of Cox *et al*. [1]. Two nodes were constrained in all three dimensions at each temporomandibular joint (TMJ). Bilateral biting was assumed since multituberculates are thought to have only employed bilateral biting [8] and rats, myomorphs with similar dental and occlusal morphology to mice, are known to chew bilaterally [9]. Therefore, one node was constrained in the axis of the bite (perpendicular to the occlusal surface) on each biting tooth on the tallest cusp, which would come into contact with food items first. For the hemimandibular models: three

nodes were constrained in all three dimensions across the surface of the condyle at the TMJ; five nodes were constrained across the mandibular symphysis in the mediolateral axis to mimic the connection with the opposing hemimandible; and a number of nodes, between one and seven, were constrained in the axis of the bite across the occlusal surface of each biting tooth. A node was constrained at the tip of each cusp on the tooth, or nodes were spaced evenly across the surface of teeth with flat occlusal surfaces, to simulate even contact with food items during biting. A variable number of nodes were constrained on the biting tooth due to the variable number of cusps on different teeth.

(iii) Input muscle forces and scaling of DigiMorph specimens

Input muscle forces from Cox *et al*. [1] were used for the squirrel, guinea pig and rat, and forces calculated herein (Tables S1-S2) were used for the mouse and *Kryptobaatar*. The CT scans used by Cox *et al*. [1] and Baverstock *et al*. [3] were obtained using iodine-based, contrast-enhanced CT. This method increases the attenuation in soft tissues through sorption of an iodine-potassium iodide (I_2KI) contrast solution, producing superior detail in muscle tissues [10]. However, since bone does not take up the contrast solution at a similar rate to soft tissues it is more difficult to distinguish boundaries between bone and muscle in resulting CT images [3], rendering automated segmentation of bone impossible. For ease of labelling in Avizo, the rat and mouse models were created using non-contrast enhanced CT scans from DigiMorph. These scans were also of specimens devoid of soft tissues making labelling much simpler. However, to use the input forces calculated from the rat and mouse specimens of Cox *et al*. [1] and Baverstock *et al*. [3] in the FE models, the DigiMorph specimens were scaled up in HyperMesh to the same mandibular

lengths as the specimens from which muscle forces were determined. The scale factors used were 1.195 for the mouse and 1.060 for the rat. The force for each muscle was divided evenly over a number of nodes (between 15 and 120) across the origin sites on the crania and attachment sites on the hemimandibles, and a vector was created in HyperMesh between the origin and attachment site of each muscle to provide the load orientation.

(c) Calculating bite force quotient (BFQ)

BFQ was calculated using the equation from Wroe *et al*. [11]:

$$BFQ = \frac{B_S}{10^{(0.6014 \times \log_{10} BoM + 1.7137)}} \times 100$$

where *BFQ* = bite force quotient, B_S = bite force (N), *BoM* = body mass (kg).

Body mass estimates were taken from the Animal Ageing and Longevity Database (AnAge: http://genomics.senescence.info/species) for extant rodents, and from Kielan-Jaworowska and Lancaster [12] for *Kryptobaatar*. BFQ was calculated for biting at the incisors and rearmost molars in all taxa, and at the fourth lower premolar for *Kryptobaatar*.

(d) Biomechanical analysis in MomentMacro

The Avizo label files of the hemimandibles and rostra were exported separately as individual TIF image stacks for each taxon, with bones and teeth labelled white on a black background to simplify thresholding. The image stacks were imported into the plugin MomentMacro v1.4B (http://www.hopkinsmedicine.org/fae/mmacro.html) in ImageJ v1.51h (National Institutes of Health, Bethesda, MD, USA) to calculate the section moduli and polar moments of inertia. Calculations were performed every 20

slices along the hemimandibles and rostra for the rat, mouse, guinea pig and squirrel image stacks and every 10 slices for the *Kryptobaatar* stacks due to the lower resolution of the scan. To correct for size discrepancies and to simply evaluate the effect of morphology, each model was rescaled to the length of the hemimandible of the rat specimen from DigiMorph by modifying the resolution of each TIF image. Scales used for each analysis (Table S8), as well as MomentMacro data from scaled analysis for the hemimandibles (Tables S9-S13) and rostra (Tables S14-S19), are provided below.

Table S8. The mandible lengths and scales used for the scaled MomentMacro analysis.

<table>
<tr><th>Specimen</th><th>Mandible length (mm)</th><th>Scale (pixels per mm)</th></tr>
<tr><td>DigiMorph rat</td><td>31.2</td><td>36.47</td></tr>
<tr><td>DigiMorph mouse</td><td rowspan="7">Scaled to same length as DigiMorph rat hemimandible</td><td>31.26</td></tr>
<tr><td>Mouse (Baverstock et al., 2013)</td><td>14.12</td></tr>
<tr><td>Squirrel (Cox et al., 2012)</td><td>15.71</td></tr>
<tr><td>Guinea pig (Cox et al., 2012)</td><td>17.71</td></tr>
<tr><td>Rat (Cox et al., 2012)</td><td>22.48</td></tr>
<tr><td>Kryptobaatar</td><td>14.00</td></tr>
</table>

Table S9. MomentMacro data from the scaled DigiMorph mouse hemimandible analysis.

Slice number	% along mandible	I_x (mm^4)	I_y (mm^4)	J (mm^4)	Z_x (mm^3)	Z_y (mm^3)	Z_p (mm^3)
93	100.00	0.001478	0.001504	0.002982	0.006511	0.006434	0.01343
100	97.49	0.04654	0.03146	0.078	0.0803	0.06553	0.1403
120	92.92	0.4972	0.22	0.7172	0.4738	0.3118	0.6929
140	88.36	1.4416	0.4086	1.8503	1.0422	0.5465	1.3612
160	83.79	2.4535	0.4807	2.9342	1.4975	0.623	1.7829
180	79.22	2.5332	0.862	3.3952	1.6453	0.9686	2.1873
200	74.66	4.1615	2.0211	6.1826	2.4294	1.5772	3.4412
220	70.09	5.769	2.8453	8.6143	3.2108	2.1286	4.2534
240	65.53	6.4383	3.0752	9.5135	3.2601	2.0785	4.5988
260	60.96	8.7126	5.0264	13.739	4.4964	3.0436	6.1456
280	56.39	56.1057	15.4599	71.5656	11.438	6.8448	13.9645
300	51.83	79.7338	18.1843	97.9181	17.1749	7.5413	20.3112
320	47.26	70.8064	15.65	86.4564	15.6866	7.1184	18.9398
340	42.69	78.3019	17.858	96.1599	19.5245	7.5968	23.9348
360	38.13	74.6568	26.6977	101.3545	16.6993	9.0309	19.1589
380	33.56	81.2609	34.4332	115.6941	13.273	9.5865	16.4379
400	29.00	110.3722	36.4508	146.8231	16.6257	9.408	19.4116
420	24.43	93.7861	26.2961	120.0821	14.1638	7.2585	16.1275
440	19.86	53.936	13.7756	67.7116	10.3637	3.4468	11.0353
460	15.30	52.8396	12.3248	65.1644	9.9518	2.9715	10.3952
480	10.73	90.5473	14.0337	104.581	14.6518	3.2677	14.4239
500	6.16	108.0504	12.8952	120.9457	18.0887	3.5578	17.6004
520	1.60	29.4966	2.77	32.2665	4.222	1.3213	4.4563
531	0.00	9.51E-05	8.13E-05	0.000177	0.000781	0.000737	0.001455

Table S10. MomentMacro data from the scaled DigiMorph rat hemimandible analysis.

Slice number	% along mandible	I_x (mm^4)	I_y (mm^4)	J (mm^4)	Z_x (mm^3)	Z_y (mm^3)	Z_p (mm^3)
249	100.00	0.000121	0.000219	0.00034	0.000943	0.001509	0.002369
270	98.86	0.05222	0.07244	0.1247	0.09804	0.1221	0.1882
290	96.96	0.1772	0.1695	0.3467	0.2368	0.2405	0.4018
310	95.06	0.3674	0.2773	0.6446	0.413	0.3782	0.6673
330	93.16	0.63	0.3782	1.0082	0.6071	0.4841	0.9161
350	91.26	1.0414	0.4526	1.494	0.873	0.563	1.2497
370	89.36	1.3584	0.5097	1.8681	1.0671	0.6131	1.3873
390	87.46	1.8349	0.5619	2.3968	1.3323	0.6517	1.5875
410	85.57	2.0437	0.5789	2.6227	1.3541	0.6564	1.6618
430	83.67	1.8378	0.5878	2.4256	1.2778	0.6721	1.6315
450	81.77	1.9418	0.6845	2.6263	1.2502	0.7501	1.5789
470	79.87	2.7853	0.8351	3.6204	1.6153	0.8649	2.0117
490	77.97	4.0763	1.0012	5.0775	2.2175	1.0257	2.6736
510	76.07	5.0286	1.572	6.6006	2.6354	1.3881	3.2297
530	74.17	5.5461	2.1687	7.7148	2.7173	1.6594	3.3581
550	72.27	5.895	2.6956	8.5906	2.986	1.931	3.7012
570	70.37	6.6372	2.9738	9.611	3.339	2.1773	4.2854
590	68.47	7.6805	3.0705	10.7509	3.9657	2.2762	4.7772

610	66.57	8.7957	3.3901	12.1858	4.497	2.5053	5.4981
630	64.67	10.1948	3.569	13.7638	4.9726	2.7481	6.1322
650	62.77	13.051	4.2869	17.3379	5.7989	3.118	6.84
670	60.87	59.1285	8.708	67.8364	9.4871	5.118	10.8608
690	58.97	113.4807	14.0396	127.5203	20.9585	7.3269	23.2461
710	57.08	131.171	16.9958	148.1668	25.2005	8.2354	28.0999
730	55.18	126.8059	17.7541	144.56	26.0538	8.5621	29.276
750	53.28	119.4616	18.1709	137.6325	25.3582	8.4903	29.0903
770	51.38	128.4187	17.8752	146.2938	26.2447	8.346	29.6549
790	49.48	124.5798	16.9484	141.5282	27.3716	8.1393	30.8708
810	47.58	112.3467	15.7083	128.055	24.9611	7.9448	28.1258
830	45.68	100.1597	15.5847	115.7444	23.0593	7.5285	24.288
850	43.78	106.2157	17.7161	123.9318	22.6687	8.3396	24.5519
870	41.88	109.6233	19.7606	129.3839	19.5042	9.5753	21.8716
890	39.98	119.2727	20.6833	139.9561	17.8566	9.8351	20.1666
910	38.08	119.5503	21.2821	140.8324	15.933	10.0278	18.1285
930	36.18	123.0654	22.6539	145.7193	15.5164	10.4556	17.912
950	34.28	133.5804	19.4269	153.0073	16.6327	8.0134	18.6693
970	32.38	144.332	13.906	158.238	18.176	6.1922	19.5508
990	30.48	137.3622	9.0834	146.4456	17.075	3.9248	17.9495
1010	28.58	89.2415	5.992	95.2335	10.3703	2.6468	10.9209
1030	26.69	66.1597	4.6999	70.8595	7.4575	2.1053	7.8914
1050	24.79	57.0622	4.1721	61.2342	9.7029	1.8857	10.3228
1070	22.89	56.4114	3.9092	60.3207	9.7165	1.7699	10.2908
1090	20.99	60.462	3.7527	64.2148	10.4684	1.6768	10.7178
1110	19.09	69.3404	3.4669	72.8073	10.9014	1.5522	11.1176
1130	17.19	77.568	2.9218	80.4899	11.3923	1.405	11.4895
1150	15.29	91.0758	2.3922	93.468	12.0469	1.2192	12.0805
1170	13.39	80.3167	1.779	82.0957	10.8546	0.9249	10.8045
1190	11.49	67.5653	1.3212	68.8865	10.2424	0.6919	10.153
1210	9.59	38.1058	0.8704	38.9763	5.6371	0.5521	5.7584
1230	7.69	5.5397	0.5486	6.0883	0.6846	0.3991	0.7521
1250	5.79	1.4624	0.3602	1.8226	0.6708	0.2979	0.8033
1270	3.89	0.8652	0.1436	1.0088	0.4878	0.1775	0.56
1290	1.99	0.28	0.03232	0.3123	0.2236	0.06352	0.2482
1302	0.00	0.000198	3.13E-05	0.000229	0.001085	0.00035	0.001414

Table S11. MomentMacro data from the scaled guinea pig hemimandible analysis.

**Slice number**	**% along mandible**	I_x **(mm⁴)**	I_y **(mm⁴)**	J **(mm⁴)**	Z_x **(mm³)**	Z_y **(mm³)**	Z_p **(mm³)**
128	100.00	0.000505	5.76E-05	0.000563	0.002146	0.000471	0.002486
130	98.98	0.07219	0.03903	0.1112	0.1076	0.07787	0.1566
150	95.58	0.3118	0.1588	0.4706	0.3489	0.2257	0.5129
170	92.18	0.5634	0.189	0.7524	0.4911	0.2709	0.6503
190	88.78	0.9701	0.3191	1.2892	0.7528	0.4324	0.9922
210	85.37	2.2991	0.6903	2.9894	1.4645	0.7357	1.8904
230	81.97	2.9379	1.4946	4.4324	1.8234	1.2332	2.7302
250	78.57	5.8648	3.4618	9.3266	3.044	2.3716	4.7866
270	75.17	10.3155	5.6207	15.9362	4.4839	3.4888	6.8136
290	71.77	10.8565	8.0952	18.9517	4.9937	4.4233	8.4502
310	68.37	9.4599	8.8994	18.3593	4.9182	4.6071	8.001
330	64.97	11.6289	17.6679	29.2968	5.7006	7.0346	9.9841
350	61.56	81.9723	91.6822	173.6545	19.7426	21.4594	33.6081
370	58.16	125.8892	147.6824	273.5716	25.3389	32.133	50.07
390	54.76	163.1843	160.4266	323.6109	34.3459	32.9482	64.3693

410	51.36	158.1934	153.0759	311.2693	31.1126	31.3441	60.0955
430	47.96	159.7898	113.5291	273.319	33.6855	25.0478	56.5675
450	44.56	118.9263	72.9301	191.8563	25.5222	18.2636	40.9944
470	41.16	123.6569	50.1672	173.824	28.8381	13.9229	40.4434
490	37.76	92.2653	37.6546	129.9199	22.8619	11.4326	30.1356
510	34.35	58.4717	22.5081	80.9798	15.6998	8.0849	20.3092
530	30.95	46.1984	12.4356	58.6341	12.6412	5.4426	14.5801
550	27.55	49.5326	12.2699	61.8024	11.8296	4.544	14.3699
570	24.15	64.32	6.4525	70.7725	11.9943	2.4203	12.118
590	20.75	93.5149	7.4351	100.95	14.1849	2.5228	14.1702
610	17.35	91.3801	7.3216	98.7017	13.7971	2.3396	13.8463
630	13.95	60.5415	5.6489	66.1903	10.7466	2.0006	10.8486
650	10.54	0.3601	0.6058	0.9659	0.2892	0.4728	0.5836
670	7.14	0.05204	0.1651	0.2172	0.07818	0.1773	0.2037
690	3.74	0.06061	0.1122	0.1728	0.09428	0.1371	0.1862
710	0.34	0.009298	0.01156	0.02086	0.02494	0.02808	0.04812
716	0.00	6.78E-06	6.78E-06	1.36E-05	9E-05	9E-05	0.000322

Table S12. MomentMacro data from the scaled squirrel hemimandible analysis.

**Slice number**	**% along mandible**	I_x **(mm^4)**	I_y **(mm^4)**	J **(mm^4)**	Z_x **(mm^3)**	Z_y **(mm^3)**	Z_p **(mm^3)**
199	100.00	0	0	0	0	0	0
200	98.72	0.001302	0.001495	0.002796	0.004646	0.005821	0.01109
220	94.43	0.4575	0.1586	0.6161	0.4228	0.2389	0.6026
240	90.15	1.4971	0.3609	1.8579	1.0225	0.4995	1.3229
260	85.87	4.0083	0.5016	4.5099	2.1009	0.6398	2.4293
280	81.58	3.763	0.6886	4.4517	1.9358	0.7811	2.3566
300	77.30	7.3286	1.706	9.0347	3.2973	1.4183	4.1419
320	73.02	13.5847	3.8586	17.4432	5.4186	2.6077	6.9588
340	68.74	22.0206	6.2957	28.3163	8.0951	3.915	10.2846
360	64.45	38.7496	8.9005	47.6501	11.6206	5.1703	14.058
380	60.17	113.4013	15.1301	128.5313	21.9463	7.8511	24.8687
400	55.89	111.9041	19.4033	131.3074	23.6727	9.3735	27.5532
420	51.61	132.0439	21.1697	153.2136	29.2097	9.5792	32.2683
440	47.32	135.3838	21.5182	156.902	30.0536	9.2696	33.4848
460	43.04	120.7213	22.8955	143.6167	28.2771	8.921	33.5608
480	38.76	131.9464	30.3723	162.3187	28.4362	11.3453	31.184
500	34.48	149.5191	29.6943	179.2134	24.508	12.664	27.8776
520	30.19	189.1745	24.4685	213.643	26.5228	11.1533	29.1464
540	25.91	170.9931	12.8933	183.8865	21.564	5.3937	22.7554
560	21.63	105.3693	9.0387	114.408	11.5033	3.6152	12.2482
580	17.34	86.7892	8.8548	95.6441	13.8304	3.2438	14.8713
600	13.06	104.8974	9.5406	114.438	16.4512	3.052	17.6361
620	8.78	203.7987	9.9209	213.7197	28.8323	3.0786	29.9852
640	4.50	84.1636	2.0975	86.2612	8.6455	1.0917	8.7798
660	0.21	0.1034	0.129	0.2324	0.1568	0.1796	0.3012
666	0.00	0.000434	0.000522	0.000955	0.002129	0.002614	0.004433

Table S13. MomentMacro data from the scaled *Kryptobaatar* hemimandible analysis.

Slice number	% along mandible	I_x (mm^4)	I_y (mm^4)	J (mm^4)	Z_x (mm^3)	Z_y (mm^3)	Z_p (mm^3)
82	100.00	0.02567	0.006607	0.03228	0.04552	0.01965	0.06219
90	95.85	2.3958	0.3501	2.746	1.4005	0.4764	1.5878
100	91.24	2.9718	0.4296	3.4013	1.6203	0.5204	1.838
110	86.64	3.7773	1.8559	5.6332	2.32	1.6051	3.2412
120	82.03	9.0725	4.6754	13.7478	4.5305	2.9797	6.5906
130	77.42	7.7403	5.3079	13.0483	4.1468	3.1774	6.413
140	72.81	22.3796	16.7202	39.0998	4.3613	5.8382	6.7705
150	68.20	70.6726	31.2334	101.906	13.184	11.4153	18.1273
160	63.59	88.1759	26.1782	114.3541	16.4773	9.767	20.612
170	58.99	102.6841	21.5245	124.2086	19.6065	8.5876	23.4575
180	54.38	108.0814	20.1372	128.2186	20.9441	8.3747	24.8014
190	49.77	102.0891	18.937	121.0261	21.2371	8.5844	25.1107
200	45.16	103.4501	19.0083	122.4585	24.4573	7.4303	28.0577
210	40.55	119.4939	26.0243	145.5182	22.5622	8.3155	23.7941
220	35.94	97.281	23.4906	120.7715	15.7026	6.9877	17.2169
230	31.34	38.125	11.5847	49.7096	6.262	4.2507	7.4846
240	26.73	15.4533	8.5547	24.008	3.8067	3.1569	5.4795
250	22.12	16.3189	10.4183	26.7373	4.6764	3.1712	7.0896
260	17.51	19.6524	10.9676	30.62	6.4687	3.0916	8.5351
270	12.90	30.3179	15.8823	46.2002	8.7112	4.2938	11.6075
280	8.29	52.815	20.4602	73.2752	14.1873	5.3941	15.8343
290	3.69	36.3208	11.9917	48.3126	10.7972	3.5823	11.3168
299	0.00	0.07003	0.01188	0.08191	0.08489	0.03102	0.0969

Table S14. MomentMacro data from the scaled DigiMorph mouse rostrum analysis.

Slice number	% along rostrum	I_x (mm^4)	I_y (mm^4)	J (mm^4)	Z_x (mm^3)	Z_y (mm^3)	Z_p (mm^3)
3	100.00	4.19E-05	0.000285	0.000327	0.000561	0.001432	0.001896
23	94.74	44.5457	7.4884	52.034	9.9012	3.1669	11.4505
43	89.47	264.6359	68.6327	333.2686	35.1245	23.1527	44.1701
63	84.21	209.6296	118.9861	328.6156	31.8494	34.7552	49.4942
83	78.95	123.0906	129.5318	252.6224	25.819	34.5085	52.0943
103	73.68	124.6691	139.4694	264.1385	27.8095	34.5915	57.5653
123	68.42	144.9074	161.6936	306.601	30.1818	39.4633	60.5249
143	63.16	199.8047	186.3581	386.1627	40.1859	44.2624	70.1873
163	57.89	246.5181	145.5809	392.0989	47.5019	33.3424	67.9691
183	52.63	262.8689	175.2828	438.1516	48.6145	26.1875	65.738
203	47.37	312.6417	399.608	712.2496	54.7876	47.095	84.0552
223	42.11	393.6662	1262.991	1656.657	65.1583	130.7617	167.3441
243	36.84	600.3048	1469.198	2069.503	88.7287	136.9266	189.7355
263	31.58	716.0286	937.2806	1653.309	91.2528	80.7635	140.4876
283	26.32	750.2075	857.5286	1607.736	82.3765	71.7698	132.7061
303	21.05	625.7154	632.553	1258.268	63.3682	51.6699	101.1507
323	15.79	672.2743	549.8331	1222.107	66.4553	44.7008	97.1668
343	10.53	729.9995	547.3529	1277.352	72.921	43.3694	98.2189
363	5.26	916.0313	602.4079	1518.439	116.4072	46.8718	108.4438
383	0.00	759.3732	619.5861	1378.959	91.329	46.9596	94.8454

Table S15. MomentMacro data from the scaled DigiMorph rat rostrum analysis.

Slice number	% along rostrum	I_x (mm^4)	I_y (mm^4)	J (mm^4)	Z_x (mm^3)	Z_y (mm^3)	Z_p (mm^3)
1	100.00	2.38E-05	7.79E-05	0.000102	0.000275	0.000496	0.000772
20	97.67	0.05288	0.9062	0.9591	0.1002	0.5681	0.5911
40	95.35	42.6903	7.4221	50.1124	9.0525	3.0257	10.6753
60	93.02	179.488	50.9927	230.4807	27.3518	18.3295	35.0185
80	90.70	301.6347	85.195	386.8297	42.624	27.6061	54.5222
100	88.37	444.8053	120.6194	565.4247	60.8643	36.0245	77.191
120	86.05	550.1849	144.1753	694.3601	76.1094	40.3395	95.8914
140	83.72	206.2115	136.6234	342.8349	34.7145	37.4228	56.9051
160	81.40	161.6351	135.7431	297.3782	32.9529	36.3831	60.5313
180	79.07	148.2603	131.5846	279.8449	32.6466	35.245	60.6985
200	76.74	146.3605	129.5138	275.8743	34.0083	35.36	62.5507
220	74.42	150.5732	134.406	284.9792	34.2929	35.9753	64.934
240	72.09	160.1037	139.8868	299.9905	35.31	37.3418	65.8059
260	69.77	175.1872	144.9169	320.1042	37.7985	38.1788	68.4658
280	67.44	190.5226	154.9656	345.4882	41.0558	39.4963	72.9954
300	65.12	208.4146	172.7446	381.1592	44.7514	41.803	78.166
320	62.79	217.6327	201.8337	419.4664	46.1711	45.2366	83.0796
340	60.47	232.2722	162.9466	395.2188	48.0887	35.3369	75.9261
360	58.14	254.2162	156.0266	410.2428	50.5005	32.5238	75.7554
380	55.81	264.9126	144.9995	409.912	49.6346	29.9582	72.0989
400	53.49	270.2214	174.1211	444.3425	49.8806	27.3827	68.6223
420	51.16	277.7817	226.904	504.6857	50.8305	32.9005	71.9079
440	48.84	318.1516	296.9789	615.1305	59.0327	40.2751	82.7436
460	46.51	340.2331	346.9193	687.1524	63.2099	45.1486	88.7591
480	44.19	358.937	445.7645	804.7015	61.7457	55.4518	100.0768
500	41.86	352.5422	669.4418	1021.984	54.1056	78.3343	119.7565
520	39.53	383.0567	912.8708	1295.928	57.6898	100.4846	142.0864
540	37.21	452.1824	1036.763	1488.945	71.7701	110.7564	158.5004
560	34.88	517.9957	1163.761	1681.757	87.9571	119.3645	171.7921
580	32.56	621.2303	1267.367	1888.598	97.829	124.3342	184.7783
600	30.23	632.8427	928.9764	1561.819	100.4181	89.2538	149.0952
620	27.91	721.7864	800.681	1522.467	104.2182	75.4473	142.9288
640	25.58	776.361	716.9435	1493.305	104.9149	66.7187	137.9084
660	23.26	812.9383	652.0978	1465.036	107.9157	59.7483	132.8945
680	20.93	908.5582	646.438	1554.996	121.4059	58.8094	138.7151
700	18.60	806.2102	581.3951	1387.605	106.6931	52.4329	121.9458
720	16.28	761.7018	551.113	1312.815	96.6559	49.1428	114.5928
740	13.95	733.8461	556.4135	1290.26	90.7807	49.1897	111.7624
760	11.63	739.5409	545.7573	1285.298	90.2551	47.8223	110.0884
780	9.30	689.2822	487.2261	1176.508	85.9256	42.0868	99.1165
800	6.98	774.502	474.8641	1249.366	96.4468	40.6885	103.7033
820	4.65	770.0105	417.4946	1187.505	106.1127	35.4186	95.4987
840	2.33	679.9932	363.3718	1043.365	92.4943	30.5513	81.7309
860	0.00	633.9319	351.3472	985.2791	86.9691	29.719	75.4597

Table S16. MomentMacro data from the scaled rat (Cox *et al*. [1]) rostrum analysis.

Slice number	% along rostrum	I_x (mm^4)	I_y (mm^4)	J (mm^4)	Z_x (mm^3)	Z_y (mm^3)	Z_p (mm^3)
20	100.00	1.57E-05	2.24E-05	3.81E-05	0.000176	0.000196	0.000491
40	96.55	0.1428	1.9554	2.0982	0.1876	0.9846	1.0138
60	93.10	35.9987	9.7768	45.7755	6.6708	3.7523	8.5524
80	89.66	201.3905	52.7121	254.1025	30.1494	18.6397	38.0358
100	86.21	386.3018	101.2789	487.5807	52.5662	33.8864	66.2734
120	82.76	572.4786	140.3157	712.7943	75.7534	42.6087	94.2167
140	79.31	282.5218	142.471	424.9929	30.3676	41.2956	45.6789
160	75.86	190.0244	141.7218	331.7462	39.5973	39.9632	68.7755
180	72.41	191.2088	156.7995	348.0083	39.2994	41.5728	72.1431
200	68.97	213.5418	174.6561	388.1978	40.8123	46.2376	74.8168
220	65.52	245.2237	195.3284	440.5521	45.7089	51.0021	82.6159
240	62.07	284.9951	229.0622	514.0573	52.1979	53.5872	93.6297
260	58.62	340.1721	213.258	553.4301	63.3883	45.3796	100.721
280	55.17	394.7525	206.8239	601.5764	70.777	37.5388	104.2233
300	51.72	437.0331	282.7725	719.8057	74.6514	42.8564	104.6665
320	48.28	470.8908	352.1847	823.0755	80.0123	48.6519	110.4627
340	44.83	516.5793	521.4153	1037.995	88.206	65.4567	129.9925
360	41.38	503.5274	820.7275	1324.255	77.1944	94.1123	151.9402
380	37.93	502.4221	1121.358	1623.78	74.157	120.2981	172.8435
400	34.48	644.6777	1443.605	2088.282	105.6617	146.0675	209.1202
420	31.03	792.3278	1466.086	2258.414	116.5997	142.8546	217.6951
440	27.59	844.6041	971.43	1816.034	119.5284	92.2162	169.4079
460	24.14	954.1741	793.6998	1747.874	124.9168	73.8247	160.3583
480	20.69	996.9571	661.8514	1658.809	126.4931	60.383	149.2003
500	17.24	1016.196	622.3166	1638.513	133.3863	56.0619	144.4117
520	13.79	979.6968	600.5442	1580.241	132.9117	53.5463	136.2166
540	10.34	948.63	520.8063	1469.436	129.9026	45.9341	124.3901
560	6.90	827.888	439.3974	1267.285	109.6119	37.8668	104.7701
580	3.45	931.791	459.7777	1391.569	115.1583	39.3441	112.2391
600	0.00	770.1442	434.4367	1204.581	96.1499	36.6301	93.1898

Table S17. MomentMacro data from the scaled guinea pig rostrum analysis.

Slice number	% along rostrum	I_x (mm^4)	I_y (mm^4)	J (mm^4)	Z_x (mm^3)	Z_y (mm^3)	Z_p (mm^3)
70	100.00	0.002886	0.000963	0.003849	0.004668	0.004187	0.006573
90	96.43	261.4551	26.503	287.9581	27.0251	13.0755	29.7514
110	92.86	721.987	46.7365	768.7234	79.5441	14.835	83.0019
130	89.29	541.2388	159.2559	700.4947	77.52	36.7374	96.8547
150	85.71	480.655	178.3533	659.0083	72.6916	36.4555	98.2529
170	82.14	426.701	191.6677	618.3687	67.8219	37.8509	96.5527
190	78.57	408.8239	244.287	653.1109	65.2784	46.6323	101.3708
210	75.00	412.7211	327.1733	739.8944	65.9499	62.1378	112.9434
230	71.43	441.4153	450.8196	892.2349	70.8961	87.1588	137.2367
250	67.86	460.5817	516.2022	976.7839	71.7187	92.0011	151.8551
270	64.29	555.929	568.6558	1124.585	82.2197	98.6137	165.3641
290	60.71	665.7328	547.7828	1213.516	102.6165	94.4465	176.8823
310	57.14	724.4471	577.2282	1301.675	114.3455	99.4007	184.8552
330	53.57	862.3837	573.5106	1435.894	125.8132	91.4052	192.6435
350	50.00	1066.378	897.6754	1964.054	131.6253	114.9603	222.5866
370	46.43	1467.596	1903.109	3370.705	161.882	192.0973	322.5345
390	42.86	1832.208	2907.584	4739.792	183.28	262.9064	404.4982

410	39.29	1988.523	4214.86	6203.383	192.8389	354.7938	498.4496
430	35.71	2365.571	5124.551	7490.122	223.9016	395.9729	576.9268
450	32.14	2844.495	5790.759	8635.254	285.3019	428.3216	636.778
470	28.57	2586.393	4834.393	7420.787	262.5917	343.3184	515.9043
490	25.00	2667.866	3917.578	6585.443	261.778	271.9259	445.7605
510	21.43	2452.576	3769.4	6221.976	239.331	253.0964	405.4178
530	17.86	2250.158	3597.252	5847.411	216.9406	235.1071	370.6729
550	14.29	2361.382	3648.36	6009.743	230.7778	231.4886	368.5371
570	10.71	2295.701	3284.811	5580.512	232.9158	204.1211	333.0773
590	7.14	1979.189	2732.558	4711.746	192.7959	167.1153	279.5081
610	3.57	1752.29	2274.113	4026.403	171.7386	138.3477	235.3412
630	0.00	1432.489	1869.713	3302.202	165.1404	114.7328	187.8588

Table S18. MomentMacro data from the scaled squirrel rostrum analysis.

**Slice number**	**% along rostrum**	I_x **(mm⁴)**	I_y **(mm⁴)**	J **(mm⁴)**	Z_x **(mm³)**	Z_y **(mm³)**	Z_p **(mm³)**
9	100.00	0.002728	0.00105	0.003778	0.01008	0.004628	0.0146
30	96.56	1.4971	18.4773	19.9744	1.2933	5.9804	6.1761
50	93.12	5.6091	48.0745	53.6836	2.4373	12.1342	11.9434
70	89.67	18.3237	85.7373	104.061	4.7576	19.3375	20.1218
90	86.23	379.7889	218.6734	598.4623	60.4882	44.2552	94.1815
110	82.79	805.2034	483.1495	1288.353	99.8779	97.7558	158.3047
130	79.35	1597.024	698.2587	2295.283	179.8255	138.7058	256.8181
150	75.90	2346.006	764.0708	3110.077	227.6547	145.9947	302.3293
170	72.46	1404.534	805.3501	2209.884	150.3932	144.8048	235.7335
190	69.02	1117.863	792.5629	1910.426	141.6457	137.9815	243.6704
210	65.58	1047.353	816.9654	1864.318	127.1356	140.0876	226.4435
230	62.13	1075.291	914.9934	1990.284	128.9057	149.5429	238.1337
250	58.69	1225.435	985.5899	2211.025	152.7443	138.53	242.2821
270	55.25	1494.03	1138.591	2632.621	183.5449	136.5819	267.5023
290	51.81	1708.963	1527.681	3236.644	201.6009	162.2857	309.5096
310	48.36	1924.937	1872.13	3797.067	230.5595	173.6451	327.2131
330	44.92	1830.959	2014.063	3845.023	208.8495	168.4448	311.3221
350	41.48	2070.007	2784.578	4854.585	220.5768	215.2848	371.9057
370	38.04	2191.303	4574.492	6765.795	216.4618	317.7336	470.5227
390	34.60	2780.351	4704.553	7484.904	265.0769	300.3537	474.7861
410	31.15	4012.11	5046.6	9058.711	353.6154	317.014	566.6118
430	27.71	5226.525	6489.636	11716.16	480.4384	397.1871	714.3453
450	24.27	5561.196	6115.455	11676.65	518.3402	357.6422	671.6355
470	20.83	5989.743	4688.192	10677.94	525.428	265.8366	599.7651
490	17.38	5227.874	3916.899	9144.772	432.8024	220.7964	495.4726
510	13.94	5401.186	3706.553	9107.739	465.7133	204.754	490.0961
530	10.50	4316.366	3436.796	7753.162	365.6204	186.3732	407.5363
550	7.06	4722.314	3774.458	8496.772	411.4521	203.5219	448.4726
570	3.61	4250.158	4011.212	8261.37	371.2707	215.621	427.6634
590	0.00	3267.277	3750.362	7017.639	302.7469	198.8558	354.6678

Table S19. MomentMacro data from the scaled *Kryptobaatar* rostrum analysis.

**Slice number**	**% along rostrum**	I_x **(mm^4)**	I_y **(mm^4)**	J **(mm^4)**	Z_x **(mm^3)**	Z_y **(mm^3)**	Z_p **(mm^3)**
6	100.00	0.006607	0.06138	0.06799	0.02268	0.08182	0.09818
10	95.10	0.1012	1.5759	1.6771	0.1691	0.9385	1.0362
20	90.20	39.4751	61.4148	100.8899	10.6973	16.0667	25.6671
30	85.29	166.0458	216.3622	382.408	42.6711	48.451	78.5624
40	80.39	453.7057	502.7599	956.4656	78.6628	93.201	159.1744
50	75.49	361.916	737.6145	1099.531	45.4023	120.7453	138.56
60	70.59	361.2372	1095.165	1456.402	79.7755	158.8397	212.7677
70	65.69	410.8668	962.541	1373.408	89.4921	126.496	180.985
80	60.78	447.6968	1007.566	1455.262	89.2478	120.0169	172.3632
90	55.88	501.9771	1332.194	1834.171	103.874	143.9796	199.5085
100	50.98	607.851	1673.377	2281.228	103.4829	162.079	222.0365
110	46.08	632.312	2014.264	2646.576	104.6341	181.2863	237.1992
120	41.18	652.6757	2130.894	2783.57	108.5079	181.3021	231.7053
130	36.27	757.627	2361.84	3119.467	120.0495	192.7495	246.5276
140	31.37	875.3652	2637.274	3512.639	128.3243	205.6354	265.7977
150	26.47	1001.418	5133.181	6134.599	147.3944	379.1267	441.3557
160	21.57	932.2033	3019.508	3951.711	145.5502	211.8363	271.2455
170	16.67	810.5445	2279.014	3089.559	126.4529	154.4664	206.4916
180	11.76	968.7076	2420.404	3389.112	140.7215	159.4175	220.8041
190	6.86	1245.915	2353.154	3599.069	178.3682	151.4252	228.5605
200	1.96	1236.959	2114.403	3351.362	169.6029	131.8192	206.6001
210	0.00	989.8609	2023.481	3013.342	155.3728	121.8101	175.6657

(e) Shapiro-Wilk test of normality for Z_x, Z_y and J data

The Z_x, Z_y and J data from MomentMacro were tested for normality using the Shapiro-Wilk test (Table S20) in SPSS Statistics 23 (IBM Corp., Armonk, NY, USA). Only the Z_x data for the rostra passed the test in each taxon, so the non-parametric Wilcoxon signed-rank test was used to test for significant differences in resistance to bending and torsion.

Table S20. Results of the Shapiro-Wilk test of normality for the Z_x, Z_y and J data, sampled every 5% along the mandibles and rostra. Those highlighted in grey passed the test (sig. >0.05) and are likely to be normally distributed.

		**Shapiro-Wilk**		
		**Statistic**	**df**	**Sig.**
**Mandible**	*Kryptobaatar* Zx	.908	21	0.051
	Mouse Zx	.874	21	0.011
	Rat Zx	.873	21	0.011
	Guinea pig Zx	.858	21	0.006
	Squirrel Zx	.873	21	0.011
	Kryptobaatar Zy	.948	21	0.309
	Mouse Zy	.881	21	0.016
	Rat Zy	.817	21	0.001
	Guinea pig Zy	.739	21	0.000
	Squirrel Zy	.893	21	0.026
	Kryptobaatar J	.872	21	0.010
	Mouse J	.842	21	0.003
	Rat J	.816	21	0.001
	Guinea pig J	.774	21	0.000
	Squirrel J	.882	21	0.016
**Rostrum**	*Kryptobaatar* Zx	.941	21	0.231
	Mouse Zx	.982	21	0.956
	DigiMorph rat Zx	.967	21	0.658
	Cox *et al*. rat Zx	.942	21	0.237
	Guinea pig Zx	.939	21	0.209
	Squirrel Zx	.953	21	0.381
	Kryptobaatar Zy	.903	21	0.040
	Mouse Zy	.825	21	0.002
	DigiMorph rat Zy	.888	21	0.021
	Cox *et al*. rat Zy	.844	21	0.003
	Guinea pig Zy	.916	21	0.074
	Squirrel Zy	.951	21	0.360
	Kryptobaatar J	.946	21	0.291
	Mouse J	.882	21	0.016
	DigiMorph rat J	.912	21	0.061
	Cox *et al*. rat J	.936	21	0.180
	Guinea pig J	.858	21	0.006
	Squirrel J	.920	21	0.086

2. Supplementary results

(a) Stress distribution supplementary figures

Figure S3. Cranial stress distribution during incisor biting for *Kryptobaatar* (A-C) and the mouse (D-F). Grey areas indicate von Mises stresses >10 MPa. Arrows indicate the biting tooth. Top row in left lateral view, middle row in dorsal view, bottom row in ventral view. Scales in B and E apply to A-C and D-F, respectively.

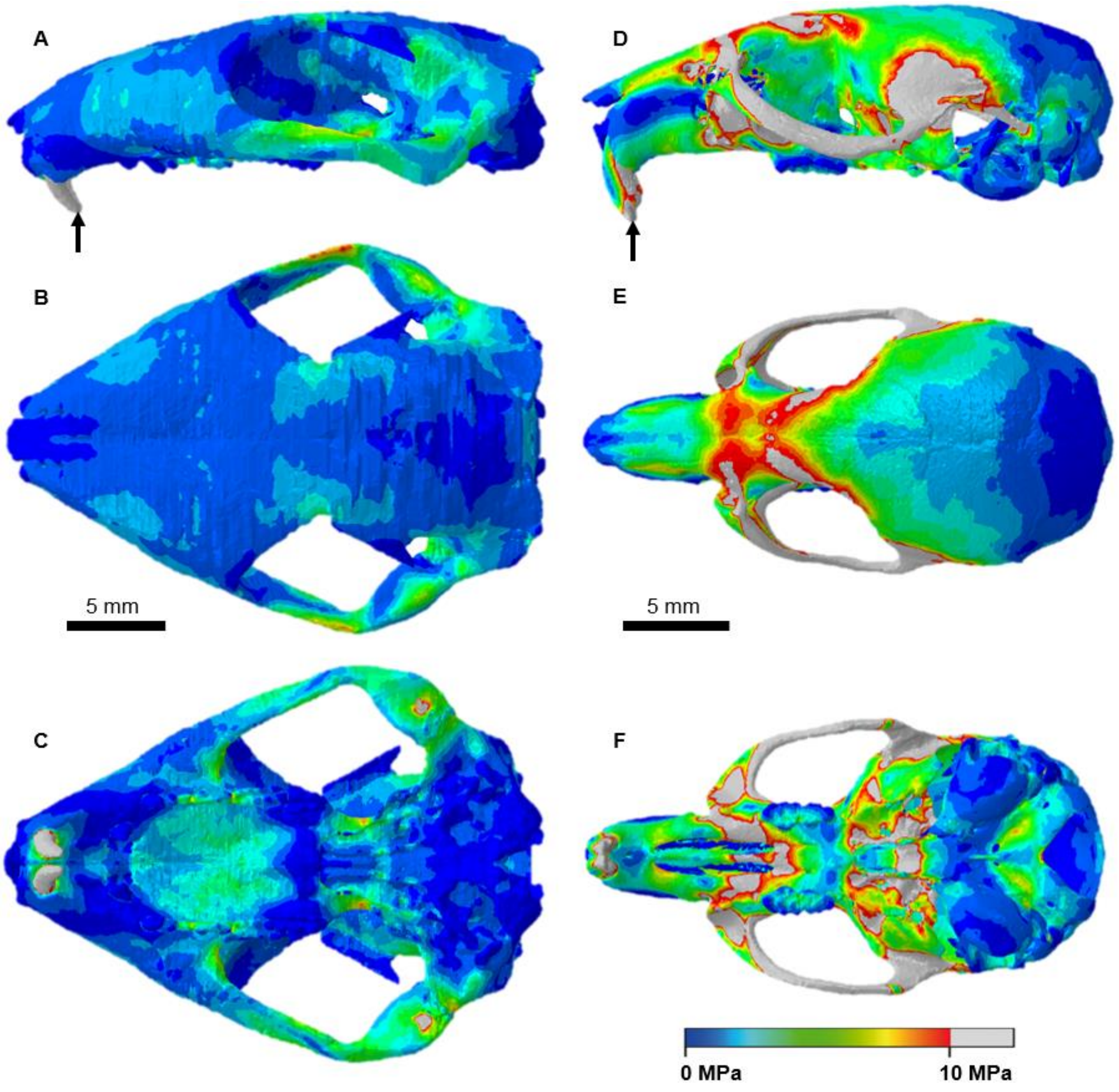

Figure S4. Cranial stress distribution during rear molar biting for *Kryptobaatar* (A-C) and the mouse (D-F). Grey areas indicate von Mises stresses >10 MPa. Arrows indicate the biting tooth. Top row in left lateral view, middle row in dorsal view, bottom row in ventral view. Scales in B and E apply to A-C and D-F, respectively.

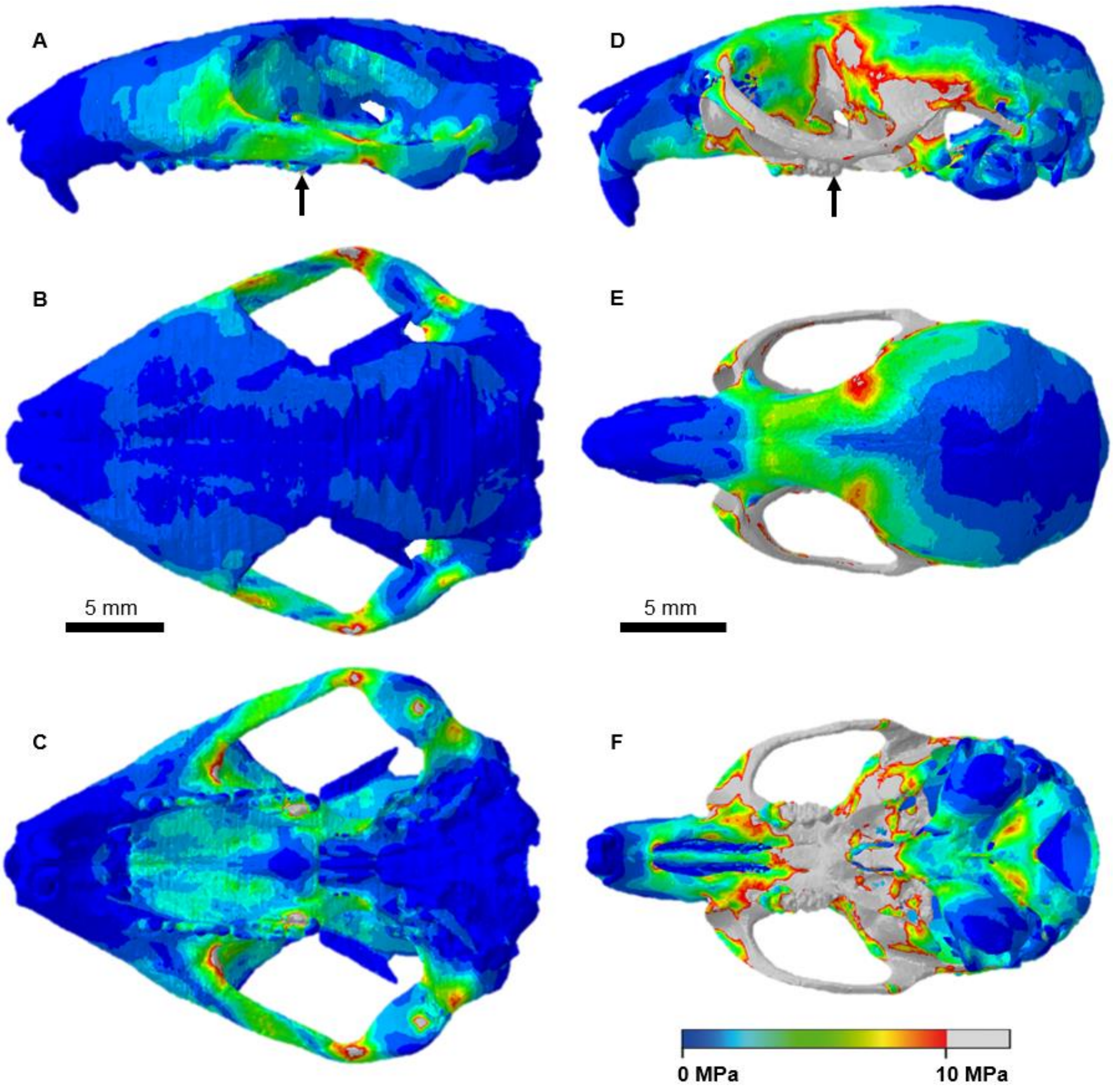

Figure S5. Stress distribution across the *Kryptobaatar* cranium during I3 (A-C) and P1 (D-F) biting. Top row in left lateral view, middle row in dorsal view, bottom row in ventral view.

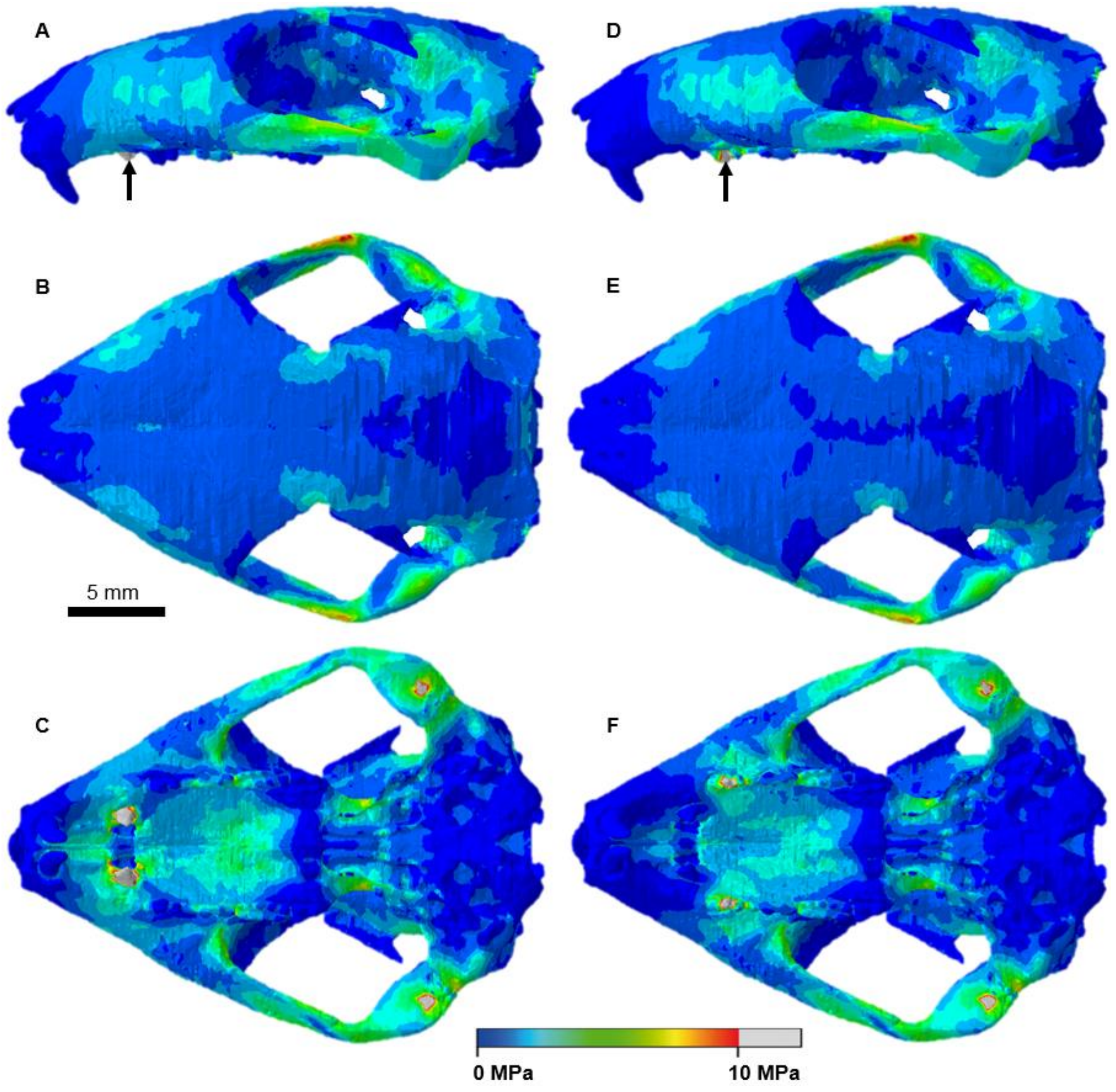

Figure S6. Stress distribution across the *Kryptobaatar* cranium during P2 (A-C) and P3 (D-F) biting. Top row in left lateral view, middle row in dorsal view, bottom row in ventral view.

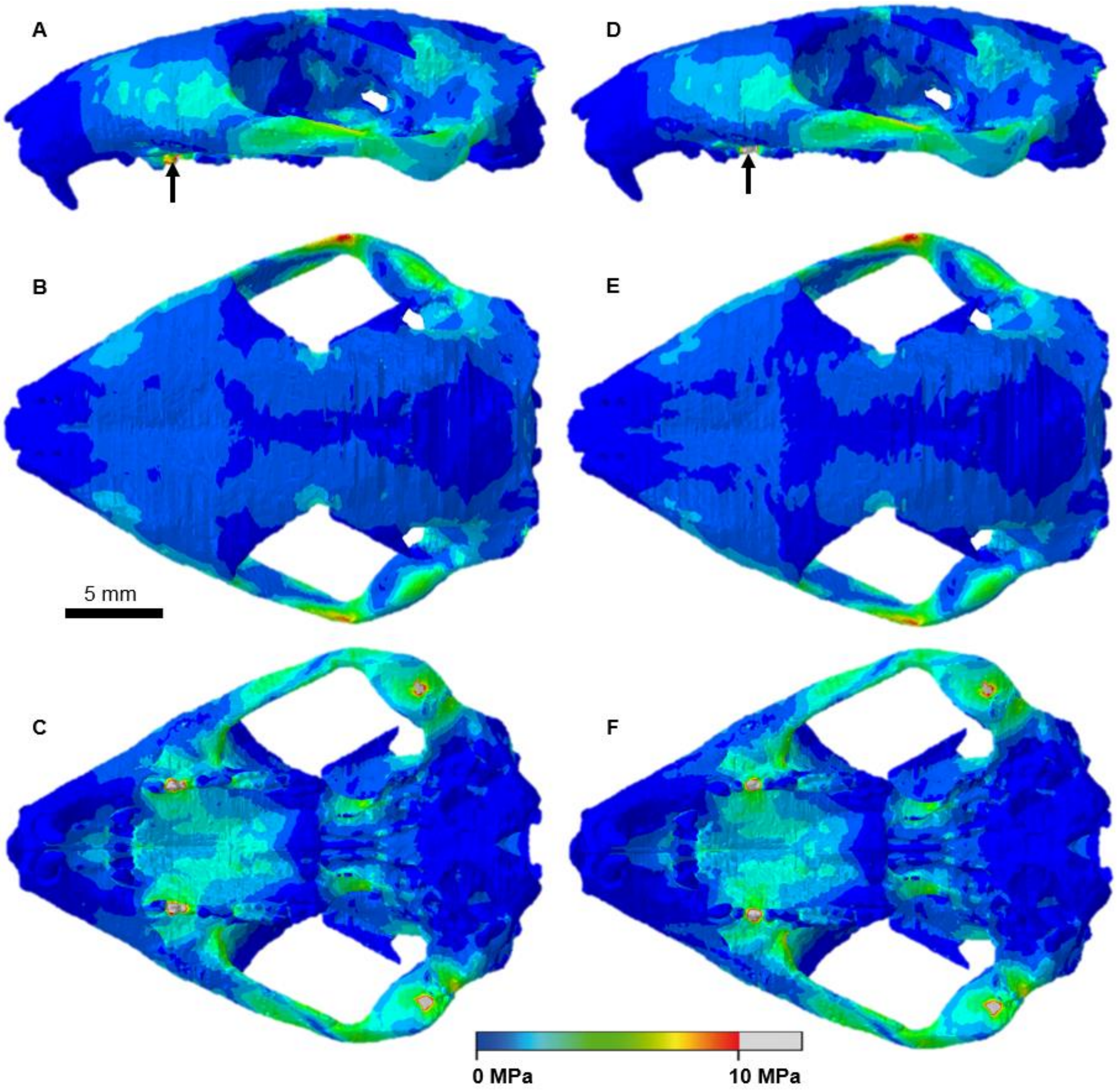

Figure S7. Stress distribution across the *Kryptobaatar* cranium during P4 (A-C) and M1 (D-F) biting. Top row in left lateral view, middle row in dorsal view, bottom row in ventral view.

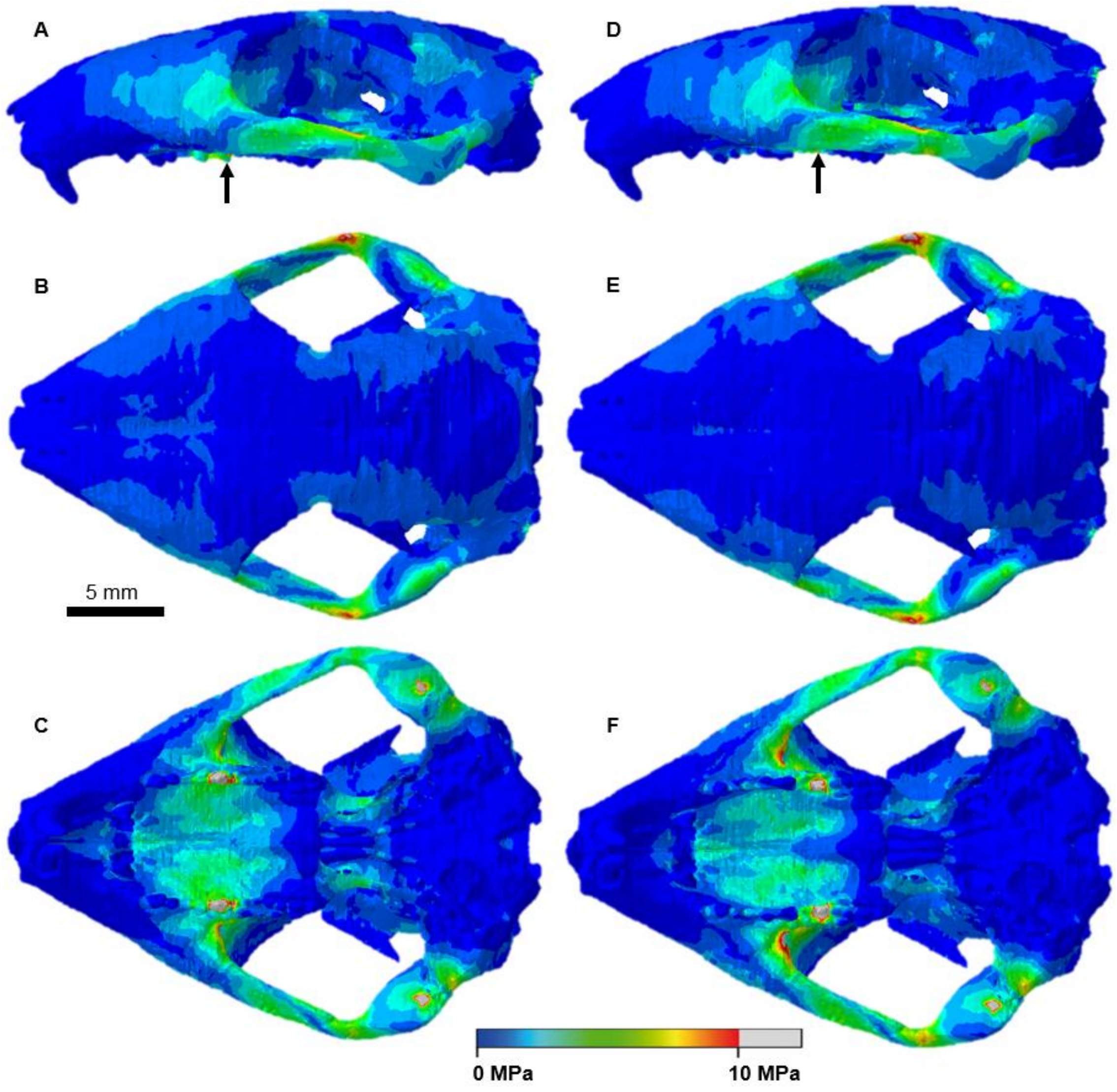

Figure S8. Stress distribution across the *Mus* cranium during M1 (A-C) and M2 (D-F) biting. Top row in left lateral view, middle row in dorsal view, bottom row in ventral view.

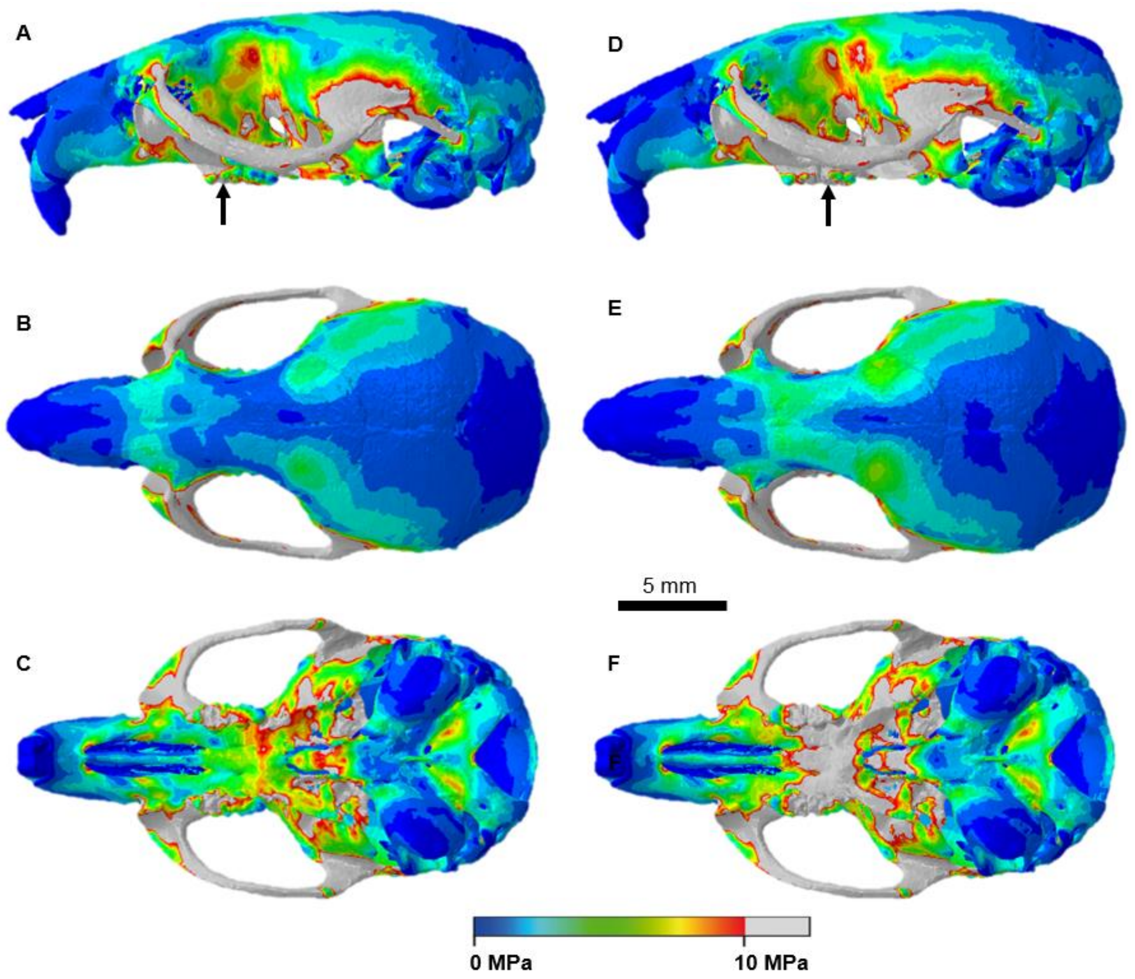

Figure S9. Hemimandibular stress distribution during incisor biting for *Kryptobaatar* (A-B), the mouse (C-D), rat (E-F), squirrel (G-H) and guinea pig (I-J). Grey areas indicate von Mises stresses >10 MPa. Arrows indicate the biting tooth. Images on the left side are in labial view, those on the right side are in lingual view. Scales on the left also apply to images on the right.

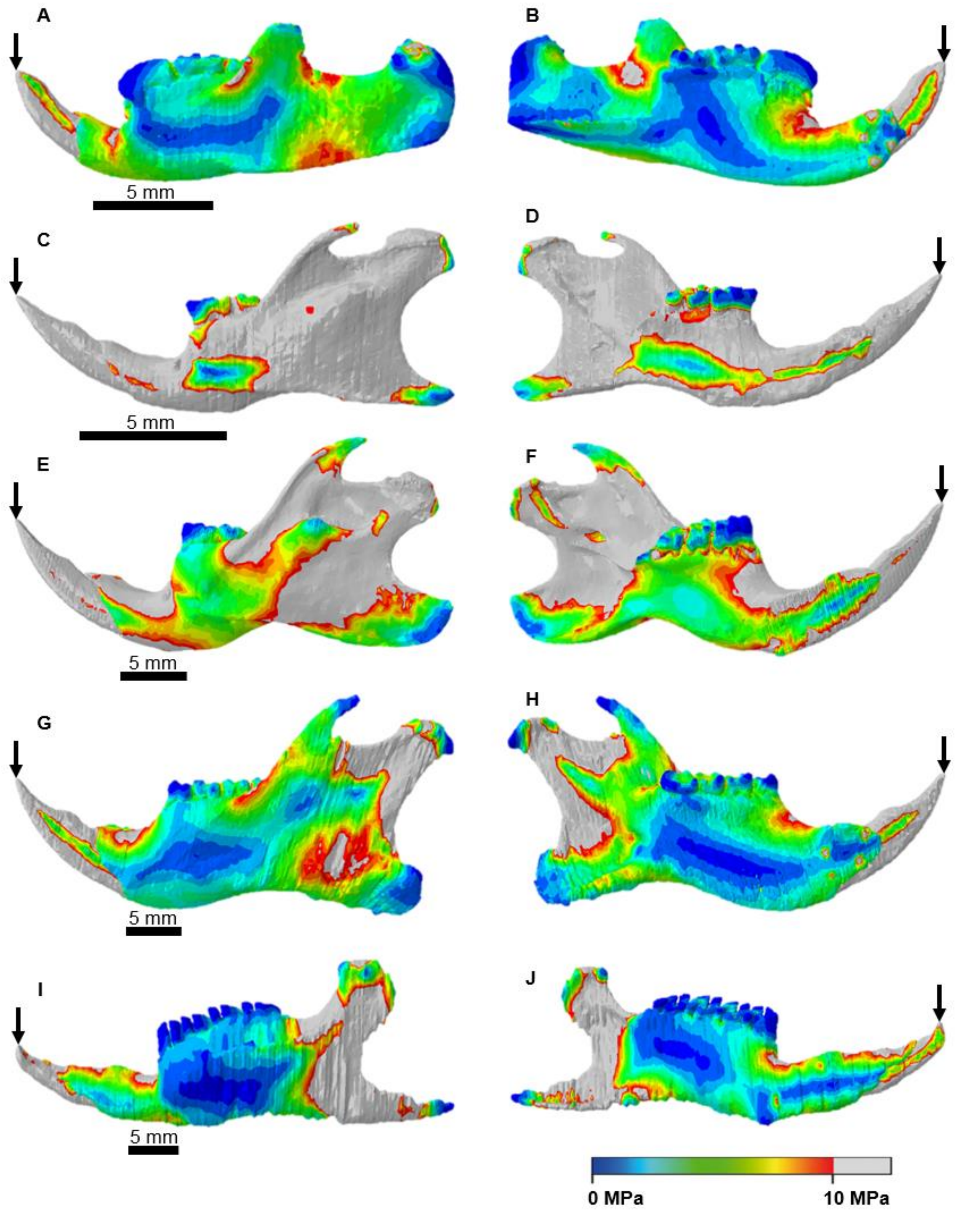

Figure S10. Stress distribution across the *Kryptobaatar* hemimandible during p4 (A-B) and m1 (C-D) biting, and across the mouse (E-H) and rat (I-L) hemimandibles during m1 (E-F, I-J) and m2 (G-H, K-L) biting. Images on the left side are in labial view, those on the right side are in lingual view.

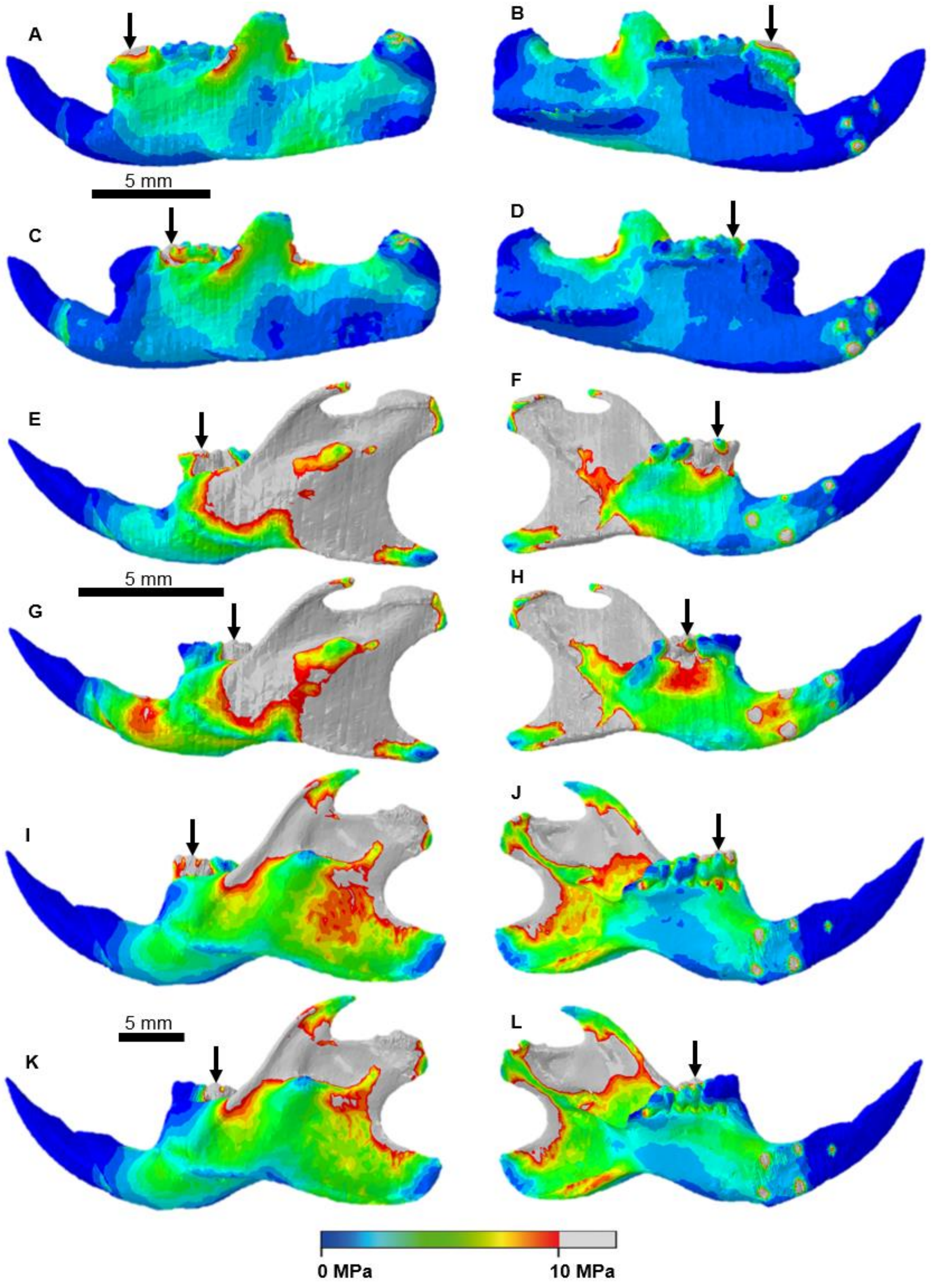

Figure S11. Stress distribution across the squirrel (A-F) and guinea pig (G-L) hemimandibles during p4 (A-B, G-H), m1 (C-D, I-J) and m2 (E-F, K-L) biting. Images on the left side are in labial view, those on the right side are in lingual view.

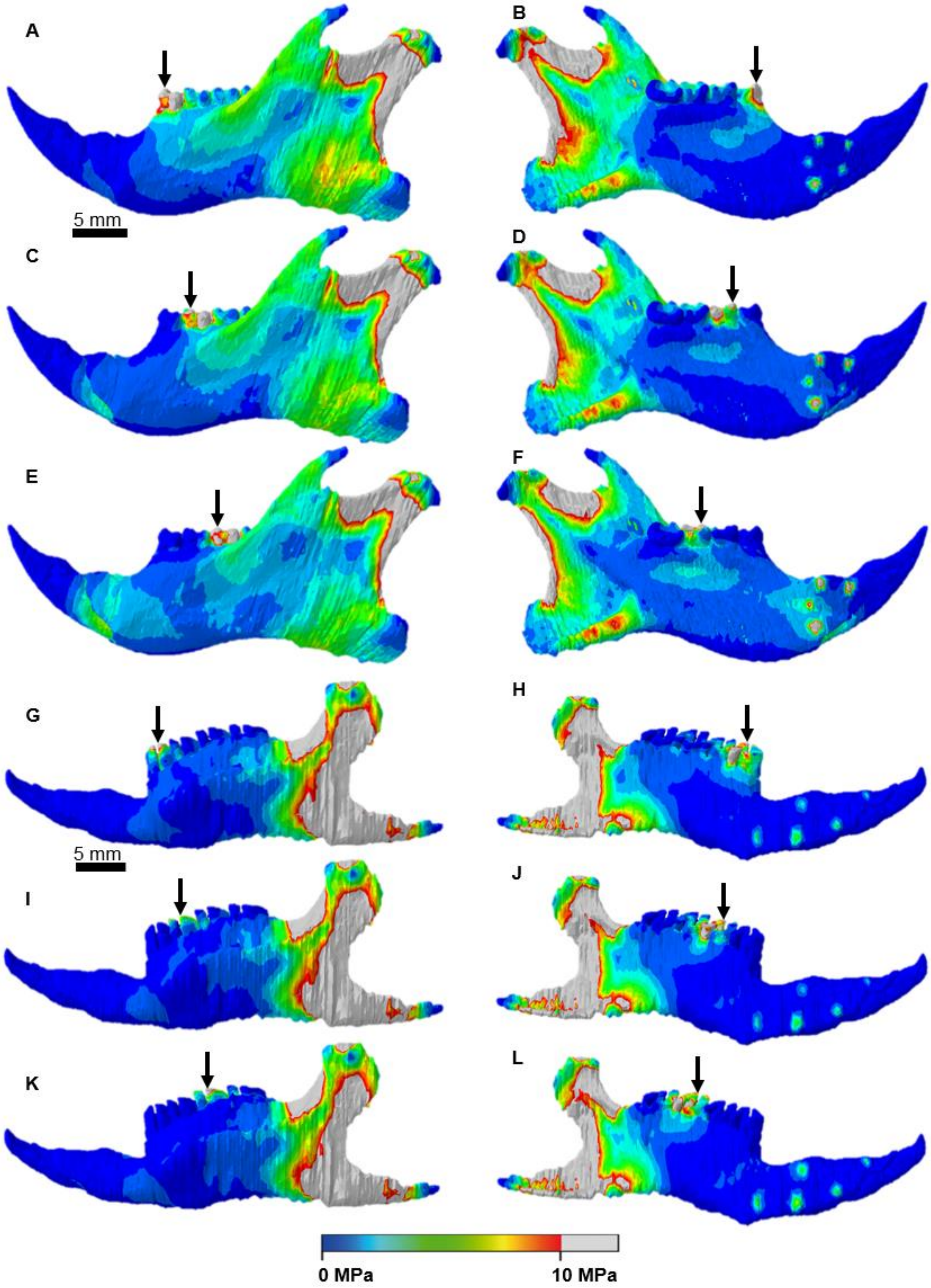

(b) Cox *et al*. (2012) cranial stress distribution figures

Figure S12. Predicted stress distribution of von Mises stresses across the crania of the squirrel, guinea pig and rat, reproduced from Cox *et al*. [1] (their figure 3). Arrows indicate the biting tooth, showing incisor bites (A-C), M1 bites (D-F) and M3 bites (G-I). Grey areas indicate von Mises stresses >10 MPa.

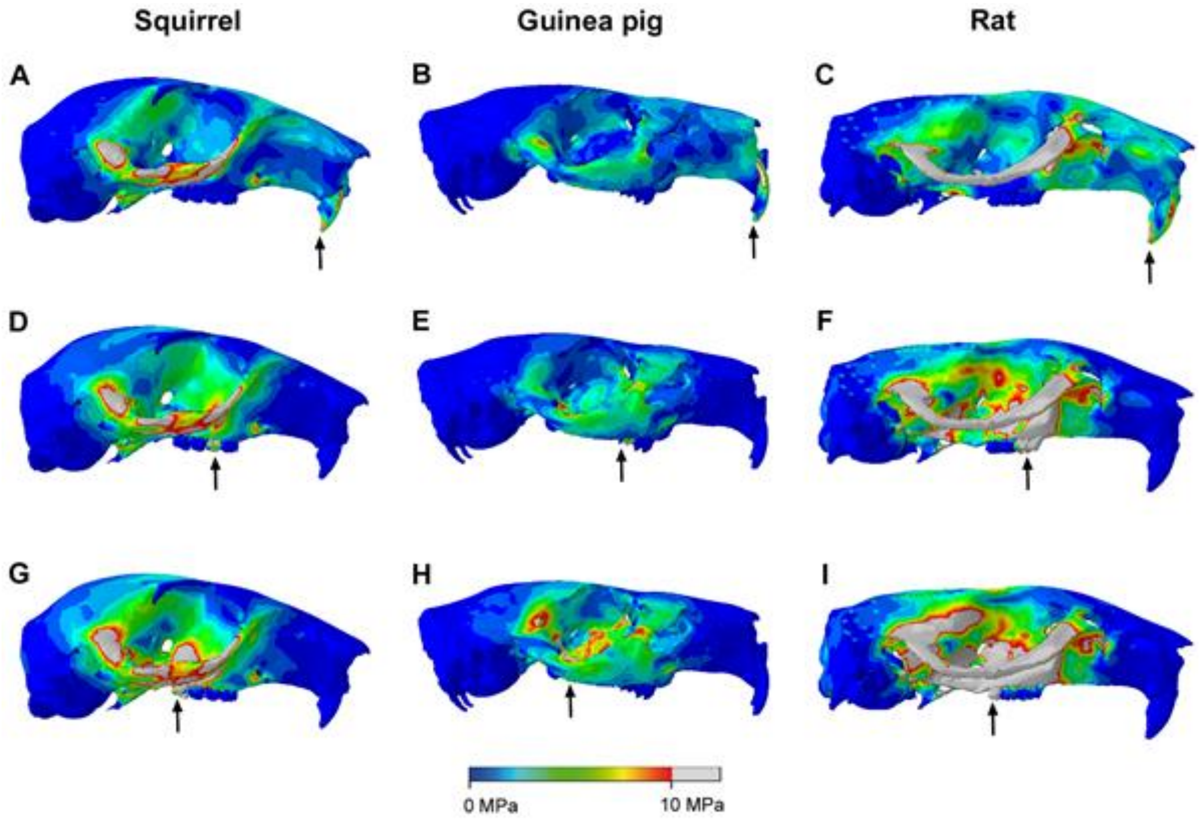

(c) Average stress data

Table S21. Raw median von Mises stress values (MPa) extracted from hemimandibular FE model outputs.

	**i1**	**p4**	**m1**	**m2**	**m3**
Kryptobaatar	2.995	1.403	1.228	1.609	-
**Mouse**	15.644	-	5.897	6.077	7.187
**Rat**	8.689	-	3.128	2.774	3.588
**Guinea pig**	2.533	1.019	0.805	0.735	0.713
**Squirrel**	4.073	1.743	1.558	1.608	2.205

Table S22. Raw median von Mises stress values (MPa) extracted from the cranial FE model output for *Kryptobaatar*.

	**I2**	**I3**	**P1**	**P2**	**P3**	**P4**	**M1**	**M2**
Kryptobaatar	1.124	1.051	0.889	0.860	0.800	0.716	0.631	0.829

Table S23. Raw median von Mises stress values (MPa) extracted from the cranial FE model output for the mouse.

	**Incisor**	**M1**	**M2**	**M3**
**Mouse**	5.027	4.312	4.865	4.956

Table S24. Calculations of percentage difference in median von Mises stress (MPa) between *Kryptobaatar* and rodent taxa for mandibular and cranial models. Cranial data for the rat, guinea pig and squirrel from Cox *et al*. [1]. +/- columns indicate directionality of differences.

**Mandible**	***Kryptobaatar***	**Mouse**	**% diff**	**+/-**	**Rat**	**% diff**	**+/-**	**Guinea pig**	**% diff**	**+/-**	**Squirrel**	**% diff**	**+/-**
i1	2.995	15.644	135.725	+	8.689	97.463	+	2.533	16.724	-	4.073	30.503	+
p4	1.403	n/a			n/a			1.019	31.682		1.743	21.579	+
m1	1.228	5.897	131.056	+	3.128	87.230	+	0.805	41.581	-	1.558	23.674	+
m2	1.609	6.077	116.251	+	2.774	53.130	+	0.735	74.610	-	1.608	0.104	-
m3	n/a	7.187			3.588			0.713			2.205		
**avg.**			**127.678**	**+**		**79.274**	**+**		**41.149**	**-**		**18.913**	**+**
**Cranium**	***Kryptobaatar***	**Mouse**	**% diff**	**+/-**	**Rat**	**% diff**	**+/-**	**Guinea pig**	**% diff**	**+/-**	**Squirrel**	**% diff**	**+/-**
Upper incisor	1.124	5.027	126.890	+	1.600	34.913	+	0.700	46.526	-	1.100	2.195	-
I3	1.051	n/a			n/a			n/a			n/a		
P1	0.889	n/a			n/a			n/a			n/a		
P2	0.860	n/a			n/a			n/a			n/a		
P3	0.800	n/a			n/a			n/a			n/a		
P4	0.716	n/a			n/a			0.500			0.700		
M1	0.631	4.312	148.968	+	1.100	54.243	+	0.550	13.658	-	0.700	10.427	+
M2	0.829	4.865	141.761	+	1.300	44.239	+	0.600	32.058	-	0.800	3.568	-
M3	n/a	4.956			1.500			0.700			0.900		
**avg.**			**139.207**			**44.465**			**30.747**			**1.555**	

(d) Mechanical efficiency data

Table S25. Mechanical efficiency raw data for the hemimandibles.

Taxon	Tooth	Reaction force at biting tooth (N)	Total input force (N)	Mechanical efficiency
Kryptobaatar	i1	2.506	13.06	0.192
	p4	3.917		0.300
	m1	5.469		0.419
	m2	8.594		0.658
Mouse	i1	5.051	20.94	0.241
	m1	13.612		0.650
	m2	14.241		0.680
	m3	16.709		0.798
Rat	i1	11.838	46.94	0.252
	m1	29.434		0.627
	m2	33.617		0.716
	m3	44.484		0.948
Guinea pig	i1	10.083	44.13	0.228
	p4	24.422		0.553
	m1	25.352		0.574
	m2	30.202		0.684
	m3	37.799		0.857
Squirrel	i1	15.190	55.20	0.275
	p4	26.459		0.479
	m1	30.301		0.549
	m2	35.683		0.646
	m3	47.550		0.861

Table S26. Mechanical efficiency raw data for the crania.

Taxon	Tooth	Reaction force at biting tooth (N)	Total input force (N)	Mechanical efficiency
Kryptobaatar	I2	4.445	26.12	0.170
	I3	5.289		0.202
	P1	6.054		0.232
	P2	6.372		0.244
	P3	6.724		0.257
	P4	7.685		0.294
	M1	9.378		0.359
	M2	12.752		0.488
Mouse	Incisor	8.617	41.88	0.206
	M1	18.845		0.450
	M2	22.910		0.547
	M3	27.532		0.657

(e) Z_x, Z_y, J supplementary data

Table S27. Wilcoxon test results for the pairwise comparisons of bending and torsional resistance along the rostrum and mandible between *Kryptobaatar* and rodent taxa, showing the Wilcoxon test statistic (*Z*) and associated *p*-value. Z_x is the dorsoventral section modulus (resistance to dorsoventral bending), Z_y is the mediolateral section modulus (resistance to mediolateral bending), *J* is the polar moment of inertia (resistance to torsion). Those highlighted in grey are significantly different. Results that shift to non-significance after applying the Bonferroni correction are marked with an asterisk (*). +/- column indicates whether rodent values are significantly higher (+) or lower (-) than *Kryptobaatar*.

	**Taxon 1**	**Taxon 2**	***Z***	***p*-value**	**+/-**
**Mandible**	**Z_x**				
	Kryptobaatar	*Mus*	-1.408	0.159	
	Kryptobaatar	*Rattus*	-0.956	0.339	
	Kryptobaatar	*Cavia*	-0.191	0.848	
	Kryptobaatar	*Sciurus*	-2.068	0.039*	+
	**Z_y**				
	Kryptobaatar	*Mus*	-1.721	0.085	
	Kryptobaatar	*Rattus*	-2.746	0.006	-
	Kryptobaatar	*Cavia*	-0.087	0.931	
	Kryptobaatar	*Sciurus*	-0.504	0.614	
	J				
	Kryptobaatar	*Mus*	-1.095	0.274	
	Kryptobaatar	*Rattus*	-0.643	0.520	
	Kryptobaatar	*Cavia*	-0.295	0.768	
	Kryptobaatar	*Sciurus*	-1.721	0.085	
**Rostrum**	**Z_x**				
	Kryptobaatar	*Mus*	-3.771	<0.001	-
	Kryptobaatar	*Rattus* (DigiMorph)	-3.458	0.001	-
	Kryptobaatar	*Rattus* (Cox *et al.*)	-3.285	0.001	-
	Kryptobaatar	*Cavia*	-3.424	0.001	+
	Kryptobaatar	*Sciurus*	-3.875	<0.001	+
	**Z_y**				
	Kryptobaatar	*Mus*	-3.841	<0.001	-
	Kryptobaatar	*Rattus* (DigiMorph)	-3.806	<0.001	-
	Kryptobaatar	*Rattus* (Cox *et al.*)	-3.875	<0.001	-
	Kryptobaatar	*Cavia*	-0.087	0.931	
	Kryptobaatar	*Sciurus*	-2.728	0.006	+
	J				
	Kryptobaatar	*Mus*	-3.806	<0.001	-
	Kryptobaatar	*Rattus* (DigiMorph)	-3.702	<0.001	-
	Kryptobaatar	*Rattus* (Cox *et al.*)	-3.702	<0.001	-
	Kryptobaatar	*Cavia*	-2.172	0.030*	+
	Kryptobaatar	*Sciurus*	-3.980	<0.001	+

References

1. Cox P.G., Rayfield E.J., Fagan M.J., Herrel A., Pataky T.C., Jeffery N. 2012 Functional evolution of the feeding system in rodents. *PLoS ONE* **7**, e36299.

2. Thomason J.J. 1991 Cranial strength in relation to estimated biting forces in some mammals. *Can J Zool* **69**, 2326-2333.

3. Baverstock H., Jeffery N.S., Cobb S.N. 2013 The morphology of the mouse masticatory musculature. *J Anat* **223**, 46-60.

4. Gambaryan P.P., Kielan-Jaworowska Z. 1995 Masticatory musculature of Asian taeniolabidoid multituberculate mammals. *Acta Palaeontol Pol* **40**, 45-108.

5. Cox P.G., Jeffery N. 2011 Reviewing the morphology of the jaw-closing musculature in squirrels, rats, and guinea pigs with contrast-enhanced microCT. *Anat Rec* **294**, 915-928.

6. Bright J.A., Rayfield E.J. 2011 The response of cranial biomechanical finite element models to variations in mesh density. *Anat Rec* **294**, 610-620. (doi:10.1002/ar.21358).

7. Cox P.G., Fagan M.J., Rayfield E.J., Jeffery N. 2011 Finite element modelling of squirrel, guinea pig and rat skulls: using geometric morphometrics to assess sensitivity. *J Anat* **219**, 696-709.

8. Wall C.E., Krause D.W. 1992 A biomechanical analysis of the masticatory apparatus of *Ptilodus* (Multituberculata). *J Vert Paleontol* **12**, 172-187.

9. Weijs W.A., Dantuma R. 1975 Electromyography and mechanics of mastication in the albino rat. *J Morphol* **146**, 1-33.

10. Jeffery N.S., Stephenson R.S., Gallagher J.A., Jarvis J.C., Cox P.G. 2011 Micro-computed tomography with iodine staining resolves the arrangement of muscle fibres. *J Biomech* **44**, 189-192. (doi:10.1016/j.jbiomech.2010.08.027).

11. Wroe S., McHenry C., Thomason J. 2005 Bite club: comparative bite force in big biting mammals and the prediction of predatory behaviour in fossil taxa. *Proc R Soc B* **272**, 619-625.

12. Kielan-Jaworowska Z., Lancaster T.E. 2004 A new reconstruction of multituberculate endocranial casts and encephalization quotient of *Kryptobaatar*. *Acta Palaeontol Pol* **49**, 177-188.
